# Stable aerobic and anaerobic coexistence in anoxic marine zones

**DOI:** 10.1038/s41396-019-0523-8

**Published:** 2019-10-17

**Authors:** Emily J. Zakem, Amala Mahadevan, Jonathan M. Lauderdale, Michael J. Follows

**Affiliations:** 1 Department of Biological Sciences, University of Southern California, Los Angeles, CA USA; 2 Department of Earth, Atmospheric and Planetary Sciences, Massachusetts Institute of Technology, Cambridge, MA USA; 3 Woods Hole Oceanographic Institution, Woods Hole, MA USA

**Keywords:** Biogeochemistry, Microbial ecology, Microbial ecology, Biogeochemistry

## Abstract

Mechanistic description of the transition from aerobic to anaerobic metabolism is necessary for diagnostic and predictive modeling of fixed nitrogen loss in anoxic marine zones (AMZs). In a metabolic model where diverse oxygen- and nitrogen-cycling microbial metabolisms are described by underlying redox chemical reactions, we predict a transition from strictly aerobic to predominantly anaerobic regimes as the outcome of ecological interactions along an oxygen gradient, obviating the need for prescribed critical oxygen concentrations. Competing aerobic and anaerobic metabolisms can coexist in anoxic conditions whether these metabolisms represent obligate or facultative populations. In the coexistence regime, relative rates of aerobic and anaerobic activity are determined by the ratio of oxygen to electron donor supply. The model simulates key characteristics of AMZs, such as the accumulation of nitrite and the sustainability of anammox at higher oxygen concentrations than denitrification, and articulates how microbial biomass concentrations relate to associated water column transformation rates as a function of redox stoichiometry and energetics. Incorporating the metabolic model into an idealized two-dimensional ocean circulation results in a simulated AMZ, in which a secondary chlorophyll maximum emerges from oxygen-limited grazing, and where vertical mixing and dispersal in the oxycline also contribute to metabolic co-occurrence. The modeling approach is mechanistic yet computationally economical and suitable for global change applications.

## Introduction

Oxygen reaches low concentrations in aquatic environments where aerobic organisms consume oxygen quickly relative to the rate of its supply [1–3]. As oxygen concentrations decline, the viable biological community generally consists of smaller organisms, and only microorganisms can efficiently utilize oxygen at the lowest (nanomolar or lower) concentrations [4, 5]. When oxygen is sufficiently depleted, metabolically diverse microorganisms use alternative electron acceptors for anaerobic respiration. In pelagic anoxic marine zones (AMZs), microbes utilize inorganic nitrogen species, producing nitrogen gas (N_2_) and the potent greenhouse gas nitrous oxide (N_2_O) [1, 4–6]. The interpretation and prediction of how these rates of fixed N loss change with warming-induced deoxygenation require appropriate descriptions of the transition from aerobic to anaerobic activity [7–9].

Observations suggest that this transition is not a sharp one. Rather, aerobic and anaerobic metabolisms seem to co-occur in AMZs [10–16]. Metagenomics and metatranscriptomics suggest widespread aerobic metabolic potential and activity throughout AMZs [13, 17]. The consumption of oxygen produced by phytoplankton at the secondary chlorophyll maximum indicates that aerobic metabolism is active in anoxic or nearly anoxic conditions [18, 19]. This co-occurring aerobic and anaerobic activity must be accounted for when predicting N loss as a function of organic matter respiration [17], since neglecting the portion of organic matter that is oxidized aerobically within AMZs overestimates N loss.

Explanations for co-occurrences typically invoke variations in ambient oxygen concentrations in time or space, such as from lateral intrusions of oxygen from equatorial jets or anoxic niches inside particles [17, 20–25]. Mixing and particle sinking can also supply immigrant cells that are adapted to different environments and that continue to metabolize upon their arrival [26–28]. However, as intervals in time and space separating sustainable aerobic and anaerobic activity become small, stable coexistence best describes community activity. Stable coexistence is supported by evidence that aerobic respiration is viable at low (nanomolar) oxygen concentrations [18, 29], and that community metabolism responds to rapid (as short as hourly) fluctuations in oxygen availability [30].

The energy that drives most metabolisms in pelagic AMZs originates directly or indirectly from primary production in the sunlit layer. Therefore, we can consider the supply of two substrates—oxygen and organic matter—as the dominant control on AMZ formation [31, 32]. To understand the chemical transformations in AMZs that impact the climate system, we must consider a diversity of N-cycling metabolisms. Two anaerobic metabolisms are responsible for the bulk of fixed N loss in the ocean: heterotrophic denitrification (${\mathrm{NO}}_3^ -$ or ${\mathrm{NO}}_2^ -$ + organic matter→N_2_O + N_2_) and chemoautotrophic anaerobic ammonia oxidation (anammox; ${\mathrm{NO}}_2^ -$ + ${\mathrm{NH}}_4^ +$→${\mathrm{NO}}_3^ -$ + N_2_) [6]. Since ${\mathrm{NO}}_2^ -$ (as well as ${\mathrm{NO}}_3^ -$) accumulates in AMZs, we can assume that heterotrophic denitrification is limited by organic matter and that anammox is limited by ${\mathrm{NH}}_4^ +$. Thus, when ${\mathrm{NO}}_2^ -$ is abundant, we may describe the transition from aerobic to anaerobic activity as the outcome of two competitions: the competition of denitrification against aerobic heterotrophy for organic matter, and the competition of anammox against aerobic ammonia oxidation for ${\mathrm{NH}}_4^ +$. The control of oxygen on these two outcomes seems to be distinct. Incubation experiments have demonstrated that anammox tolerates higher oxygen concentrations than denitrification [30]. This is consistent with observations in the Bay of Bengal, where anammox, but not denitrification, was measured at intermediate oxygen concentrations (10–200 nM) [33].

However, understanding the transitions of this complex network of metabolisms along an oxygen gradient remains incomplete. Though observations and model estimates of ${\mathrm{NO}}_2^ -$ production and consumption rates reveal excessive ${\mathrm{NO}}_2^ -$ supply [16, 34–36], reasons for the differing rates remain unclear. Accumulation to concentrations beyond those limiting any metabolism distinguishes this secondary ${\mathrm{NO}}_2^ -$ maximum from the primary ${\mathrm{NO}}_2^ -$ maximum, which may constitute the limiting subsistence concentration for aerobic ${\mathrm{NO}}_2^ -$ oxidizing microorganisms [37]. Also, why is anammox a viable metabolism at higher oxygen concentrations than denitrification [30]? One explanation for inhibition of anaerobic metabolism is the oxygen sensitivity of specific enzymes [30]. An alternative perspective is that microbial community function forms, and is formed by, the chemical potential of the environment, and that specific enzymatic machinery has consequently evolved over time in response to this chemical potential. Thus, chemical potential may explain the energetic favorability of metabolism at a more fundamental level.

Here, we explore the coexistences and competitive exclusions among the diverse O_2_- and N-cycling metabolisms that emerge when metabolisms are related more directly to chemical potential. Ideally, a model of microbial metabolism would anticipate biogeochemical function by estimating the activity of microorganisms from first principles—as a function of underlying chemical and physical constraints—allowing for universal applicability. We work towards this ideal by constructing a microbial ecosystem model where metabolic functional types are described by underlying redox chemistry, by theoretically and empirically determined efficiencies, and by parameterizations of resource uptake grounded in cell physiology. Unlike models that rely on imposed oxygen inhibition concentrations and other AMZ-specific measured parameters [38, 39], this allows for a self-consistent model that has no information about AMZ metabolic biogeography as input, and can then be compared with observations. Moreover, resolving interactions of metabolic functional types obviates the need for prescribed oxygen thresholds and allows for the emergence of stable coexistence of competing metabolisms.

Specifically, we use Resource Ratio Theory [40] to first develop and demonstrate a theory for sustained coexistence with an idealized example of aerobic and anaerobic heterotrophic populations competing for organic matter. We consider this competition as either actual competitive interactions between two distinct obligate aerobic and anaerobic populations or an internal cellular process of a facultatively anaerobic population that switches its electron acceptor to maximize its growth rate in a dynamic environment. Second, we develop a redox-based parameterization of a set of diverse microbial metabolisms crucial to AMZ O_2_ and N cycling, and examine the outcome of their interactions in a simplified system (a virtual chemostat). Finally, since physical processes as well as biological processes shape AMZs, we incorporate the metabolic model into a two-dimensional (2D) circulation that captures the essential physical features giving rise to AMZ formation. The model, expanded to include phytoplankton and zooplankton functional types, represents a fully dynamic microbial ecosystem spanning oxic to anoxic states. In the idealized AMZ, coexistence is sustained in the oxycline from vertical mixing as well as at an emergent secondary chlorophyll maximum.

## Materials and methods

### Theory: stable coexistence despite competition

#### Metabolic model

We develop a theoretical model of two metabolisms. Each requires two resources: electron donor *S* and electron acceptor *X*. An equation for the growth rate *μ*_*i*_ (t^−1^) of a population associated with each metabolism *i* can be formulated from the specific rate of uptake *V* (mol resource mol biomass^−1^ t^−1^) of each required resource and the yield *y* (mol biomass mol resource^−1^) associated with each resource. Using Leibig’s Law of the minimum, the limiting growth rate is

1 $$\mu _i = \min \left[ {y_{S_i}V_{S_i}(S_i),y_{X_i}V_{X_i}(X_i)} \right],$$

where uptake is a function of the resource concentration and may be represented with a saturating (Michaelis–Menten) form or as limited by diffusive supply, for example. The two metabolisms may also be considered as occurring (“competing”) within one facultative population that can use multiple electron acceptors.

We can anticipate the outcome of a competitive interaction between the two populations by comparing the population-specific subsistence concentrations of that resource [40]. Here we derive and compare the expressions for the subsistence concentrations of organic matter for populations carrying out aerobic and anaerobic heterotrophy. We describe organic matter uptake with a Michaelis–Menten form with specific maximum uptake rate *V*_*maxOM*_ and half-saturation constant *K*_*OM*_, and then combine the organic matter-limited growth rate (*y*_*OM*_*V*_*OM*_) with a steady state balance in which growth rate equals the specific loss rate *L* (t^−1^), which may represent mortality, maintenance costs, consumption by grazers, or viral lysis. Solving for the organic matter concentration OM^*^ gives

2 $$OM_i^ \ast = \frac{{K_{{OM}_i}L_i}}{{y_{{OM}_i}V_{{maxOM}_i} - L_i}}.$$

The kinetic parameters for organic matter uptake are uncertain and should vary with substrate and with population, but if we assume that uptake kinetics for the same substrate may be optimized similarly for different organisms adapted to similar conditions (which must be the case for a facultatively aerobic population), and if we assume that specific loss rates are also similar (such as if the populations are subject to the same predation rate), the subsistence organic matter concentrations differ predominantly by the yield $y_{OM_i}$ in the denominator. Free energies of reactions predict that O_2_ is a superior electron acceptor to ${\mathrm{NO}}_3^ -$ across a wide range of activities [41, 42], and thus that the aerobic organic matter yield ($y_{OM_O}$) is greater than the anaerobic ($y_{OM_N}$) for the same electron donor. With $y_{OM_O}$ > $y_{OM_N}$, the aerobic subsistence concentration is lower than the anaerobic subsistence concentration ($OM_O^ \ast < OM_N^ \ast$) and an aerobic population can competitively exclude the anaerobic population when both are limited by the same organic substrate. This simply anticipates the dominance of aerobic metabolism in the oxygenated biosphere.

Analogously, the aerobic population becomes oxygen-limited once O_2_ is depleted to its subsistence concentration of oxygen, ${\mathrm{O}}_2^ \ast$ [43, Table 2]. If DIN supply is reduced, the anaerobic population would also become limited by its electron acceptor at ${\mathrm{NO}}_3^{ - \ast }$ (or ${\mathrm{NO}}_2^{ - \ast }$).

#### The threshold for stable aerobic and anaerobic coexistence

Following previous work [40, 44, 45], we derive an expression that determines whether the simulated aerobic heterotroph competitively excludes the anaerobic heterotroph or whether the two coexist as a function of the relative supply rates of organic matter (*OM*_*in*_) and oxygen (O_2*in*_) in a chemostat where growth rate *μ*_*i*_ equals dilution rate *D*. In Appendix 1, we provide the details of the derivation, which considers the condition allowing both aerobically and anaerobically sustained biomass in the steady state balances for organic matter and oxygen (Eqs. (T3) and (T4) in Table 1). We call this threshold *ϕ*:

3 $$\phi = \frac{{D({\mathrm{O}}_{2in} - {\mathrm{O}}_2^ \ast )}}{{D(OM_{in} - OM_O^ \ast )}}r^{ - 1},$$

where *r* is the ratio of oxygen to organic matter demand of the aerobic heterotrophic metabolism: $r = y_{OM_O}y_{\mathrm{O}_2}^{ - 1}$ (mol O_2_ utilized per mol *OM* utilized; Eq. (A11)). If *ϕ* > 1, more oxygen is supplied than is required to aerobically consume all of the organic matter supplied, and the anaerobic metabolism can be competitively excluded. If *ϕ* = 1, oxygen and organic matter are supplied in the exact ratio demanded by the aerobic metabolism. If *ϕ* < 1, more organic matter is supplied than can be processed aerobically, and the excess organic matter can be metabolized anaerobically. Thus, *ϕ* = 1 is the threshold below which aerobic and anaerobic metabolism can stably coexist. The aerobic metabolism is never competitively excluded (as long as oxygen remains an energetically favorable electron acceptor).

**Table 1:** Equations for the model with two metabolic functional types—aerobic heterotroph (*B*_*O*_) and anaerobic heterotroph (*B*_*N*_)—in a virtual chemostat

$$\frac{{dB_O}}{{dt}} = \underbrace {B_O\left( {\mu _{O}} \right.}_{{\mathrm{biomass}}\,{\mathrm{synthesis}}}\left. { - D} \right)$$	(T1)
$$\frac{{dB_N}}{{dt}} = \underbrace {B_N\left( {\mu _{N}} \right.}_{{\mathrm{biomass}}\,{\mathrm{synthesis}}}\left. { - D} \right)$$	(T2)
$$\frac{{dOM}}{{dt}} = \underbrace {D\left( {OM_{in}} \right.}_{{\mathrm{supply}}}\left. { - OM} \right) - \underbrace {\frac{1}{{y_{OM_{O}}}}\mu _{O}B_O - \frac{1}{{y_{OM_{N}}}}\mu _{N}B_N}_{{\mathrm{organic}}\,{\mathrm{matter}}\,{\mathrm{consumption}}}$$	(T3)
$$\frac{{d{\mathrm{O}}_2}}{{dt}} = \underbrace {D\left( {\mathrm{O}_{2in}} \right.}_{{\mathrm{supply}}}\left. { - \mathrm{O}_2} \right) - \underbrace {\frac{1}{{y_{\mathrm{O}_2}}}\mu _{O}B_O}_{{\mathrm{oxygen}}\,{\mathrm{consumption}}}$$	(T4)
$$\frac{{dN}}{{dt}} = \underbrace {D\left( {N_{in}} \right.}_{{\mathrm{supply}}}\left. { - N} \right) + \underbrace {\left( {\frac{1}{{y_{OM_{O}}}} - 1} \right)\mu _OB_O + \left( {\frac{1}{{y_{OM_{N}}}} - 1} \right)\mu _NB_N}_{{\mathrm{remineralization}}} - \underbrace {\frac{1}{{y_N}}\mu _NB_N}_{{\mathrm{denitrification}}}$$	(T5)

Growth rates of the two populations (*μ*_*O*_ and *μ*_*N*_) are calculated with Eq. (1). To correctly estimate the consumption of both limiting and nonlimiting substrates, uptake is written in terms of the growth rate as $V_j = y_j^{ - 1}\mu$ for substrate *j*. For organic substrate, (1 − *y*_*j*_)*V*_*j*_ is partitioned towards respiration (remineralization) rather than biomass synthesis. Biomass *B*, organic matter *OM*, and dissolved inorganic nitrogen (*N*) are resolved in concentrations of nitrogen

In an AMZ, where organic matter supplies nearly all chemical energy, *ϕ* can broadly delineate the locations of anaerobic activity. However, the incoming and outgoing fluxes of oxygen and organic matter cannot be easily decomposed as in the chemostat (where the incoming flux $F_{{\mathrm{O}}_2in} = D{\mathrm{O}}_{2in}$), and so we use an approximate form of *ϕ*. When it is feasible for both oxygen and organic matter substrates to be depleted to low concentrations relative to supply, one can neglect the outgoing fluxes (i.e., $F_{{\mathrm{O}}_2out} = D{\mathrm{O}}_2^ \ast \approx 0$ and $F_{OM_{out}} = D(OM^ \ast ) \approx 0$), but the sinking of particulate organic matter makes some of the organic matter unavailable to the microbial community at a given depth. In this context, a more useful approximation to Eq. (3) takes into account the divergence of the organic matter flux ($\nabla \cdot OM = F_{OM_{in}} - F_{OM_{out}}$) as

4 $$\phi _{ocean} = \frac{{F_{{\mathrm{O}}_2in}}}{{\nabla \cdot OM}}r^{ - 1}.$$

Here, *OM* represents the sum of both dissolved and sinking (particulate) organic matter supply, though we would expect the sinking portion to contribute to the majority of the outgoing flux. Ratio *r* is similar to the “respiratory quotient,” or the amount of CO_2_ produced per mol O_2_ consumed [46], although it additionally reflects the amount of organic matter assimilated into biomass. The value of *r* appropriate for the ocean is a function of an average heterotrophic growth efficiency given the complex soup of organic substrates (Eq. (A11), Fig. A1). It converges to the value of the respiratory quotient for the observed low efficiencies [46], and decreases if the average efficiency increases substantially (such as could happen with “fresh” organic matter input following a bloom). A decrease in *r* increases *ϕ*, in which case less O_2_ supply is required to maintain strictly aerobic activity. Using this balance, locations where *ϕ*_*ocean*_ < 1 can be identified as energetically favorable for anaerobic metabolisms. In other words, anaerobic activity is sustainable once oxygen supply is low enough that the ratio of available oxygen to organic matter is lower than the ratio of demand.

### Redox-based description of metabolic functional types

A minimum set of microbial metabolisms (in addition to oxygenic photoautotrophy) mediates the climatically relevant N-cycling in and around anoxic zones [1, 6]. For each metabolism, we relate the main N-based substrates and excretion products to biomass *B* in units of N as: aerobic heterotrophy (*OM* + O_2_→*B*_*HetO*_ + ${\mathrm{NH}}_4^ +$), heterotrophic nitrate reduction to nitrite (*OM* + ${\mathrm{NO}}_3^ -$→$B_{HetNO_3}$ + ${\mathrm{NH}}_4^ +$ + ${\mathrm{NO}}_2^ -$), heterotrophic denitrification of nitrite (*OM* + ${\mathrm{NO}}_2^ -$→$B_{HetNO_2}$ + ${\mathrm{NH}}_4^ +$ + N_2_), heterotrophic dissimilatory nitrate (or nitrite) reduction to ammonium (DNRA: *OM* + ${\mathrm{NO}}_3^ -$→*B*_*HetDNRA*_ + ${\mathrm{NH}}_4^ +$), chemoautotrophic aerobic ammonia oxidation (${\mathrm{NH}}_4^ +$ + O_2_→*B*_*AOO*_ + ${\mathrm{NO}}_2^ -$), chemoautotrophic aerobic nitrite oxidation (${\mathrm{NO}}_2^ -$ + O_2_→*B*_*NOO*_ + ${\mathrm{NO}}_3^ -$), and chemoautotrophic anammox (${\mathrm{NH}}_4^ +$ + ${\mathrm{NO}}_2^ -$→*B*_*anx*_ + ${\mathrm{NO}}_3^ -$ + N_2_).

**Fig. 1:**
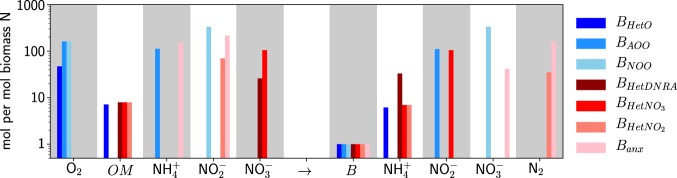
Estimates of the stoichiometries of substrate demand (yield *y*^−1^) and excreted products relative to synthesis of biomass *B* for the metabolic functional types (three aerobic in shades of blue, and four anaerobic in shades of red): aerobic heterotrophy (*B*_*HetO*_), ammonia oxidation (*B*_*AOO*_), nitrite oxidation (*B*_*NOO*_), dissimilatory reduction of nitrate to ammonium (*B*_*HetDNRA*_), nitrate-reducing heterotrophy ($B_{HetNO_3}$), denitrifying heterotrophy ($B_{HetNO_2}$), and anaerobic ammonia oxidation (anammox; *B*_*anx*_). Currency for generic organic matter substrate *OM* and biomass *B* is moles of N

Following established methodology [47], we describe these metabolisms as metabolic functional types by combining electron-normalized half-reactions to form the catabolic and anabolic full reactions for each (Appendix 2). Electron fraction *f* then partitions the electron flow towards biomass synthesis vs. respiration for energy. This results in a whole-organism stoichiometry that quantifies the amount of each substrate required to provide the electrons and elements for synthesis of one unit of N-based biomass (ex: 112 mol ${\mathrm{NH}}_4^ +$ per mol *B*_*AOO*_ for aerobic ammonia oxidation; Fig. 1). We consider this amount of required substrate in terms of a substrate-specific yield (ex: $y_{NH_{4B_{AOO}}} = 112^{ - 1}$), and so we can represent each metabolism in general form as

5 $$\frac{1}{{y_{S_i}}}S_{red,i} + \frac{1}{{y_{X_i}}}X_{ox,i} \to B_i + \left( {\frac{1}{{y_{S_i}}} - 1} \right)S_{ox,i} + \frac{1}{{y_{X_i}}}X_{red,i},$$

where *S* (mol N L^−1^) is an electron donor substrate and *X* (mol N L^−1^) is the electron acceptor in reduced (*S*_*red*_, *X*_*red*_) or oxidized (*S*_*ox*_, *X*_*ox*_) form, and *B* (mol N L^−1^) is the biomass of type *i*. See Appendix 2 for a detailed description of each metabolism and Fig. 1 and Table A1 for the resulting stoichiometries and yields.

Redox chemistry links the yields to electron fraction *f*, which we constrain using a combination of theoretical and empirical analysis (Appendix 2 and Table A1). For the chemoautotrophic metabolisms, after comparing anammox stoichiometry with previous analysis of aerobic nitrification [37, 47, 48], we assigned the same value of *f* = 0.03 to anammox as well as to the nitrifiers to most robustly test the competition between them (Appendix 2). The resulting stoichiometry suggests that anammox is less efficient than aerobic ${\mathrm{NH}}_4^ +$ oxidation with respect to ${\mathrm{NH}}_4^ +$, requiring about 150 vs. 110 mol ${\mathrm{NH}}_4^ +$ per mol biomass N, but more efficient than aerobic ${\mathrm{NO}}_2^ -$ oxidation with respect to ${\mathrm{NO}}_2^ -$, requiring about 220 vs. 330 mol ${\mathrm{NO}}_2^ -$ per mol biomass N. For all heterotrophs, we describe growth on an average pool of marine organic matter, and so we assigned an average marine bacterial growth efficiency to the aerobic heterotroph ($y_{OM_{HetO}} = {\mathrm{0.14}}$) [46], which corresponds to *f* ≈ 0.1 (Appendix 2). Informed by free energies as above, we assigned a slightly lower organic matter yield to all three anaerobic heterotrophs (10% lower). We assigned them equal organic matter yields because we do not here consider the further characteristics that determine the outcome of interactions among anaerobic heterotrophic metabolisms [49, 50]. With this simplification, differences in the amount of electron acceptor required by each anaerobic heterotroph in Fig. 1 reflect only the stoichiometries of the electron-normalized redox reactions. We vary these values in an ensemble of model solutions (Appendix 3).

Table 2 represents a matrix of all possible interactions in the model. Since the complex network prevents any simple prediction of the outcome of the interactions, the diagnostic framework provided by subsistence (R^*^) concentrations is a useful way to interpret model solutions. We list the R^*^ concentrations for each metabolism and substrate calculated with the yields, uptake parameters, and the chemostat dilution rate. For all functional types, we assume one uptake parameterization for organic matter, another for DIN, and a third for oxygen (Table A2). This essentially assumes a similar cell size and proteome allocation for all populations, and that differences in uptake kinetics do not determine the outcome of any competition. This assumption is justified if populations competing for the same substrate in the same environment have similarly optimized their enzyme allocation, and that tradeoffs exist between traits, such as substrate affinity, maximum growth rate, and defense strategies. However, the framework does allow for the possibility of (and interpretation of) alternative outcomes if differences in these parameters do consistently distinguish populations carrying out competing metabolisms in ways that change their relative subsistence concentrations.

**Table 2:**
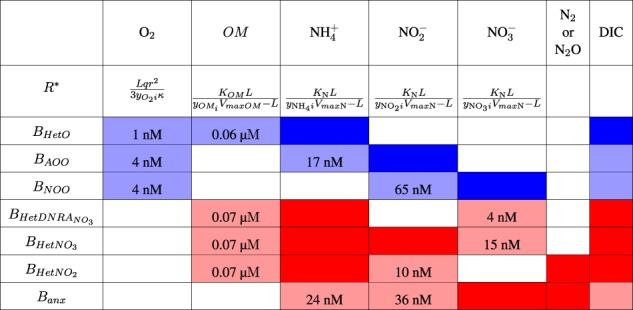
The interaction matrix of the metabolic functional types (blue: aerobic metabolisms, red: anaerobic metabolisms)

Lighter colored squares indicate required substrates, and are overlaid with the associated subsistence (R^*^) concentration calculated with loss rate *L* as the virtual chemostat dilution rate (*D* = 0.05 per day). Darker colored squares indicate waste metabolic products. For each column, all organisms requiring that substrate (all lighter colored squares in the column) can potentially compete with one another, while each pair of dark and light colors indicates the potential for a syntrophic (cross-feeding) interaction. DIC consumption and production is also noted. Yields and uptake kinetic parameters are listed in Tables A1 and A2

## Results

### The transition from competitive exclusion to stable coexistence

We first examine the competition of aerobic and anaerobic metabolism in a simulated chemostat to make two points: (1) that the theoretical prediction of the metabolic shift is borne out and (2) that the same shift is predicted whether associated with a reorganization of community metabolism by balancing the populations of obligate types, or by facultative readjustment of individual metabolisms. We examine two parallel models, one with distinct (obligate) aerobic and anaerobic populations, and a second with a facultative population.

The set of ordinary differential equations in Table 1 describes the biomass *B* of the populations associated with the distinct metabolisms, as well as organic matter, oxygen, and DIN. In the parallel simulation with the facultative population, one bulk biomass *B*_*fac*_ carries out whichever metabolism yields a higher growth rate at each time step ($\mu _{fac} = \max (\mu _{O},\mu _{N})$). We assume abundant DIN availability (*N*_*in*_ = 30 μM) as in the mesopelagic ocean, giving solutions independent of DIN.

**Fig. 2:**
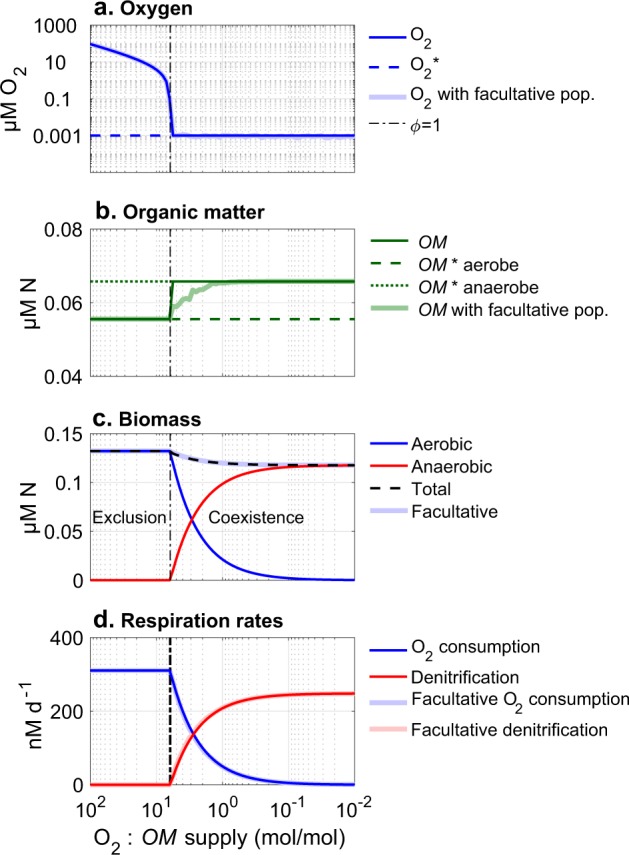
Steady state solutions for two metabolisms—aerobic and anaerobic heterotrophy—in a virtual chemostat for a varying ratio of O_2_ to organic matter (*OM*; mol N) supply. Two parallel simulations are illustrated: one with discrete aerobic and anaerobic heterotrophic metabolic functional type populations, and one with a single facultatively anaerobic population. Respiration rates for the facultative population indicate the time-averaged use of O_2_ and DIN. The vertical dashed black line indicates *ϕ* = 1, the threshold at which anaerobic metabolism becomes sustainable

We examine the equilibrium solutions to the equations for a varying ratio of oxygen to organic matter supply by varying the incoming oxygen concentration (Fig. 2). In the model with the two obligate types, the anaerobic type is competitively excluded at a high supply ratio (abundant oxygen) without a prescribed oxygen inhibition because its organic matter subsistence concentration is higher than that of the aerobe ($OM_N^ \ast > OM_O^ \ast$). When the supply ratio decreases below threshold *ϕ* (black vertical line), the aerobic metabolism becomes limited by oxygen and is only able to oxidize a fraction of the available organic matter, and the anaerobic metabolism is sustained.

Throughout this domain of coexistence, oxygen is maintained at the subsistence concentration of the aerobe (${\mathrm{O}}_2^ \ast$; Fig. 2a). Organic matter is maintained at the higher subsistence concentration of the anaerobe ($OM_N^ \ast$; Fig. 2b), which is qualitatively consistent with observations of decreased attenuation of the particulate organic matter flux in low oxygen environments (i.e., reduced particulate consumption) [51, 52]. The ratio of aerobic to anaerobic biomass and associated respiration rates decreases proportionally with the relative decrease in oxygen supply (Fig. 2c, d). This pattern is consistent with observations of aerobic and anaerobic bacterial biomass competing for sulfide at varying oxygen to sulfide ratios [53].

The solution with the facultative population shows nearly identical results (lighter colored lines in Fig. 2). The oxygen concentration wavers slightly due to the synchronous switching from aerobic to anaerobic growth, and the organic matter concentration is between $OM_N^ \ast$ and $OM_O^ \ast$ for the period at which the facultative population utilizes both oxygen and DIN at similar fractions over time.

The model shows that: (1) anaerobic sustainability is a predictable function of the relative supply of oxygen and an electron donor such as organic matter, (2) the ratio of aerobic to anaerobic activity reflects this relative supply, (3) aerobic activity can be sustained at ${\mathrm{O}}_2^ \ast$ where O_2_ is supplied, such as through photosynthesis or physical transport, and (4) a facultative population model gives a nearly equivalent biogeochemical outcome to a model with two obligate populations at steady state.

### Diverse N-cycling metabolisms in a chemostat

We next examine the equilibrium state of the interactions of the diverse N-cycling metabolic functional types as a function of oxygen and organic matter supply. To consider the uncertainty in the parameterizations, we computed an ensemble of solutions for which the parameter values dictating the yields (and thus also ratio *r*) were sampled randomly from plausible ranges of uncertainty (Equations and detail in Appendix 3).

**Fig. 3:**
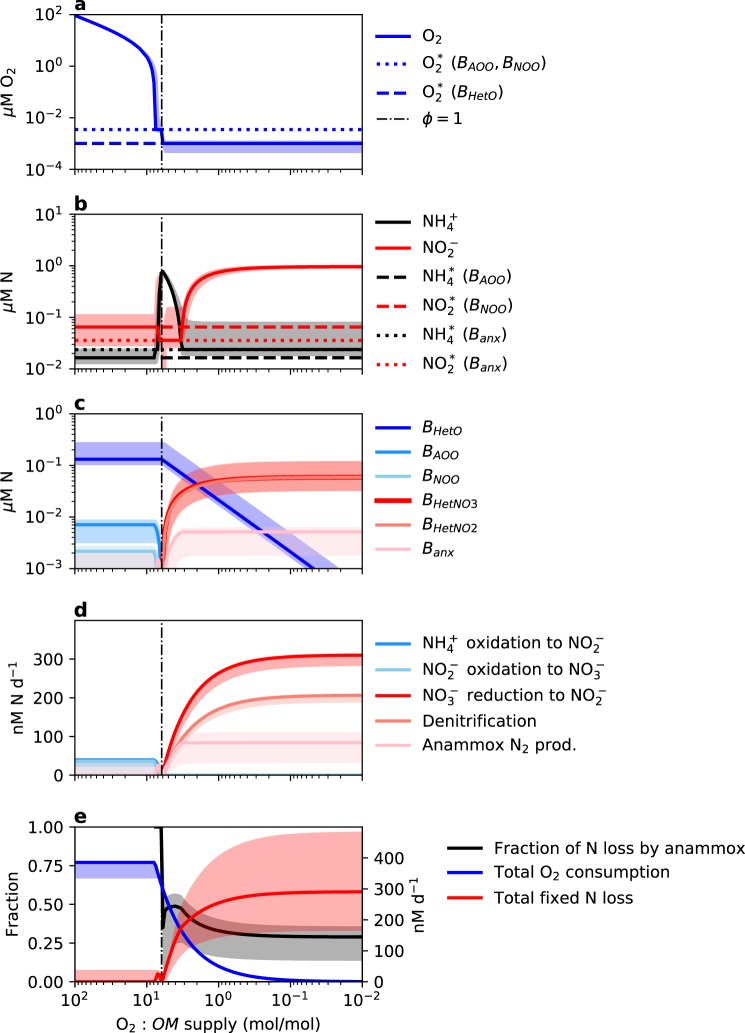
Steady state solutions for the diverse O_2_- and N-cycling metabolic functional types in a virtual chemostat for a varying ratio of O_2_ to organic matter (*OM*; mol N) supply. The shaded region indicates the 5th–95th percentile of the ensemble of solutions. Incoming *OM* concentration is 1 μM N

We illustrate the model solutions without DNRA in Fig. 3. When the DNRA functional type is included, ${\mathrm{NH}}_4^ +$ but not ${\mathrm{NO}}_2^ -$ accumulates in the anoxic state (Fig. A3), which is consistent with some observations, but not with the characteristic state of AMZs [14]. We conclude that DNRA is likely less efficient at utilizing organic matter than the other anaerobic heterotrophic metabolisms, and that low or sporadic rates of DNRA must be sustained by a process not resolved in the current model, such as time-varying blooms of organic input (see Appendix 3 for further discussion).

#### Oxygenated state

At high oxygen supply (O_2_:*OM* ≳ 10 mol/mol in Fig. 3), the three aerobic metabolisms coexist syntrophically. All anaerobic metabolisms are competitively excluded, except for some simulations in the ensemble in which the ${\mathrm{NH}}_4^ +$ yield of anammox is similar to or higher than that of aerobic ammonia oxidation (Fig. 3d, e). Concentrations of ${\mathrm{NH}}_4^+$ and ${\mathrm{NO}}_2^ -$ are maintained at the subsistence concentrations of the aerobic ${\mathrm{NH}}_4^+$-oxidizing and ${\mathrm{NO}}_2^ -$-oxidizing populations, respectively [37] (Fig. 3b). This simulates microbial community function in oxygenated environments below the euphotic zone.

#### Anoxic state

At the lowest relative oxygen supply rates (O_2_:*OM* /≲ 1 mol/mol in Fig. 3), O_2_ is depleted to ${\mathrm{O}}_2^ \ast$, and all anaerobic metabolisms are sustained. [${\mathrm{NH}}_4^ +$] is maintained at the subsistence concentration of the anammox population, while [${\mathrm{NO}}_2^ -$] accumulates higher than any subsistence concentration (Fig. 3b). The accumulation results from the imbalance in ${\mathrm{NO}}_2^ -$ supply and demand reflecting the stoichiometries of the redox reactions. Normalized by electron transfer, 1.5 times more ${\mathrm{NO}}_3^ -$ is reduced to ${\mathrm{NO}}_2^ -$ by the ${\mathrm{NO}}_3^ -$-reducing population than ${\mathrm{NO}}_2^ -$ reduced by the denitrifying population. Anammox also consumes ${\mathrm{NO}}_2^ -$, but not enough to counter the imbalance since its growth is limited by ${\mathrm{NH}}_4^ +$. This perhaps explains the formation of the secondary ${\mathrm{NO}}_2^ -$ maximum.

As oxygen supply decreases, the rates of denitrification and anammox converge to a constant ratio. The fraction of anammox contribution to total fixed N loss is 29% (Fig. 3e), which is consistent with the theoretically and empirically observed fraction of about 30% [6, 30, 32, 54]. This fraction remains constant across variations in yields and uptake parameters. A lower anammox yield translates to lower annamox-associated biomass, for example, but the water column rate remains the same, demonstrating that bulk water column rates are ultimately determined by organic matter supply.

The aerobic heterotroph remains the only active aerobic metabolism in the anoxic state. Once its growth is oxygen-limited, it competes against the nitrifiers for oxygen. The nitrifiers are excluded because they demand more O_2_ for growth (lower $y_{\mathrm{O}_2}$) and thus have a higher ${\mathrm{O}}_2^ \ast$ (Fig. 3a, blue dashed lines). This higher O_2_ demand reflects the energetic cost of C fixation for chemoautotrophy compared with the average heterotroph. However, in real environments, the heterogeneity of organic matter and diversity of the heterotrophic community should result in a diversity of populations each associated with a distinct ${\mathrm{O}}_2^ \ast$, and the portion of the community with higher ${\mathrm{O}}_2^ \ast$ may be excluded at higher oxygen supply than the nitrifiers. These results do suggest, however, that a subset of aerobic heterotrophs can subsist in the anoxic core of AMZs, which is consistent with genetic and transcriptomic evidence [17].

#### Intermediate state

In between the oxic and anoxic end-member states (O_2_:*OM* ≈ 1–10 mol/mol in Fig. 3), the simple model predicts a complex intermediate state. Oxygen supply is just low enough to limit the growth of the nitrifiers, but not yet low enough to limit the aerobic heterotroph, which continues to be limited by organic matter supply. Because of this, oxygen is maintained at the nitrifiers’ higher ${\mathrm{O}}_2^ \ast$ (Fig. 3a), and, analogous to the heterotrophic case study in Fig. 2, their oxygen-limited growth allows for residual reduced DIN that then sustains anammox. This represents a distinct threshold for the onset of anaerobic anammox, matching experimental results [30]. In the model, anammox is a sustainable metabolism at a higher oxygen supply than denitrification because it competes against the chemoautotrophic nitrifiers for DIN. Chemoautotrophic nitrification demands significantly more O_2_ than heterotrophy to synthesize the same amount of biomass (Fig. 1), and so the oxygen supply that becomes limiting for them is higher than the oxygen supply that becomes limiting for the average heterotroph. Nitrate reduction and denitrification do not become energetically favorable until aerobic heterotrophy is limited by oxygen, and anammox is ${\mathrm{NO}}_2^ -$-limited until N_2_ has accumulated (Fig. 3b). Thus, the redox-based model anticipates the adaptation of anammox clades to tolerate higher concentrations of oxygen to exploit available chemical potential.

### A two-dimensional idealized AMZ

The virtual chemostat model provided an organized framework for interpreting the ecology of the diverse O_2_- and N-cycling metabolisms in and around AMZs in isolation from the impacts of ocean circulation. However, physical supply and dispersal are important characteristics of the real system. As a final study, we included transport by incorporating the ecological model into an idealized circulation model.

**Fig. 4:**
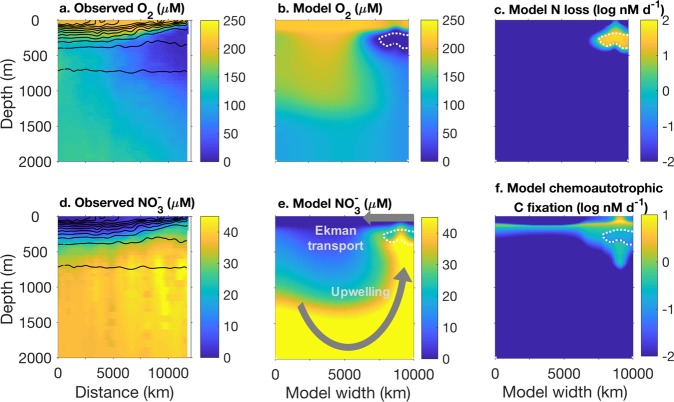
Observations and model simulations of the 10° S transect across the Pacific Ocean. Observations of O_2_ (**a**) and ${\mathrm{NO}}_3^ -$ (**d**) are from the World Ocean Atlas 2013 climatology [65], with distance from 175° longitude in the W. Pacific. Arrows in **e** indicate the idealized 2D flow field, with intensified upwelling driven by wind-stress-induced Ekman transport. Steady state solutions of the metabolic ecosystem model incorporated into the flow field include: O_2_ (**b**), fixed N loss from heterotrophic denitrification and anammox (**c**), ${\mathrm{NO}}_3^ -$ (**e**), and chemoautotrophic C fixation calculated from biomass production rates with a biomass C:N of 5 (**f**)

A 2D overturning circulation qualitatively simulates the 10°S transect across the S. Pacific basin (Fig. 4). A closed flow field with a width of 10,000 km and a depth of 2000 m is forced with wind stress mimicking the climatological mean (Fig. A4; see Appendix 4 for equations and detail). A mixed layer is prescribed with an attenuating vertical diffusivity coefficient, and eddy stirring is represented with a horizontal diffusivity constant. The resulting flow field simulates Ekman transport, “eastern” coastal upwelling, and dispersed downwelling in the “west” (Fig. 4e).

The six microbial metabolic functional types in Fig. 3 are advected and diffused by the circulation, along with two phytoplankton types and three zooplankton grazing types that produce and consume oxygen, respectively. (Equations, detail, and parameter values in Appendix 4 and Table A3). Zooplankton grazers consuming phytoplankton are inhibited at low oxygen concentrations. Vertical migration of zooplankton preying on the non-photosynthetic microbes is simulated by allowing them to consume microbial biomass throughout the anoxic zone and accounting for their oxygen consumption above and below [55–57]. (See Appendix 4 for detail.) Thus total zooplankton activity is reduced, but does not cease, in the AMZ. Two pools of organic matter—one sinking and one non-sinking—are sourced from the mortality of all populations. Oxygen fluxes across the air–sea interface. Fixed N lost to denitrification or anammox is summed and distributed as a source of ${\mathrm{NO}}_3^ -$ equally over the domain at all depths, simulating distant N fixation.

**Fig. 5:**
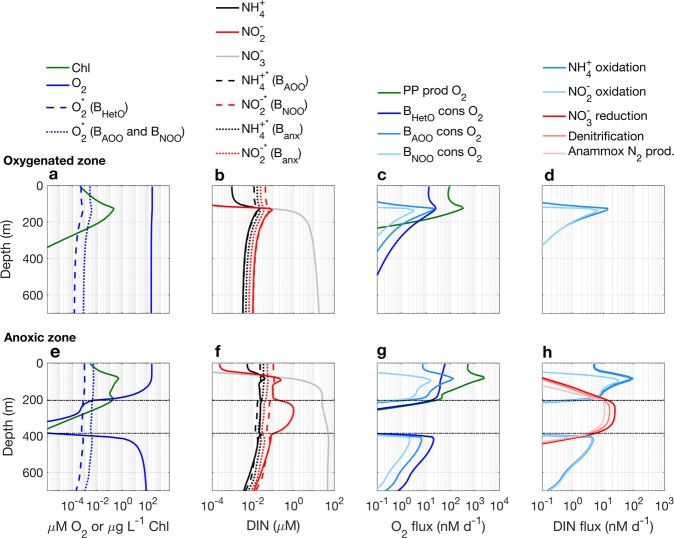
Steady state solutions through two water columns in the 2D model: one in the “western” oxygenated zone and one in the anoxic portion of the “eastern” upwelling zone. The vertical dashed and dotted lines indicate subsistence concentrations (R^*^s). The horizontal black dashed lines (*ϕ*_*ocean*_ = 1) indicate the zone of stable anaerobic activity, which does not account for transient coexistence due to dispersal from vertical mixing

The solutions demonstrate how the combination of circulation and surface productivity—due to intrinsic links between intensified coastal upwelling and a lack of ventilation below in eastern boundaries [58]—results in anoxic zone formation (Figs. 4 and 5; complete solutions in Figs. A5 and A6). Intensified upwelling enhances primary productivity, which produces more sinking organic matter input to the unventilated zone below where aerobic respiration depletes oxygen. Figure 5 illustrates profiles through two water columns: one in the oligotrophic, oxygenated zone, where only the aerobic metabolisms are sustained (Fig. 5a–d), and one through the anoxic zone where all metabolisms are sustained at various depths (Fig. 5e–h).

The characteristic profiles through the oxygenated zone resulting from the metabolic model have been previously discussed [37]. Here we highlight aspects of the modeled anoxic zone, their parallels with observations, their mechanisms in the model, and implications for AMZ biogeochemistry:

#### AMZ formation is controlled by relative rates of oxygen and organic matter supply

In the simulated anoxic zone, oxygen is depleted to nanomolar concentrations or lower, with the lowest values due to dispersal of aerobic cells into the anoxic zone. Immigrant cells can drive down the limiting resource below the subsistence concentration of a purely local population because immigration makes up for the reduced local growth implied by the lower resource concentration [27]. The modeled vertical profile simulates the sharp oxycline characteristic of pelagic anoxic zones (Fig. 5e) [4]. Modeled ${\mathrm{NO}}_3^ -$ has the characteristic concave profile that indicates N loss (Fig. 5f). The fraction of anammox contribution to total fixed N loss increases at the periphery of the AMZ (Fig. A7), although this intermediate state is less pronounced than in the chemostat model due to the mixing. Some organic matter sinks below the anoxic zone, fueling a deep aerobic community for an additional few hundred meters (Fig. 5g, h). The solution state can be anticipated by calculating *ϕ*_*ocean*_ using the model flow field. The contour *ϕ*_*ocean*_ = 1 (the dashed lines in Figs. 4 and 5) delineates—to first order—the domain in which anaerobic metabolism is sustainable. In Fig. A2, we plot resulting O_2_ consumption, N loss, and O_2_ concentrations against the incoming supply of O_2_ and organic matter to each grid box, which further demonstrates the resource ratio control.

#### Vertical mixing sustains anaerobic metabolisms in oxygenated waters

In the model, significant rates of all three anaerobic metabolisms are sustained at very high (tens of μM) oxygen concentrations above the anoxic zone (Fig. 5h). Since *ϕ*_*ocean*_ does not account for transport, the N loss outside the dashed line *ϕ*_*ocean*_ = 1 (and some of the oxygen consumption within, as mentioned above) is a consequence of the dispersal of biomass. This may be a mechanism supporting the high diversity of metabolisms in the oxycline in real AMZs [59, 60].

Dispersal-driven anaerobic respiration remains when including the facultative type in the 2D model: as in Fig. 2, with respiration rates averaged over time, the steady state solutions with facultative aerobic heterotrophs are indistinguishable. When obligate aerobic, obligate anaerobic, and facultative heterotrophs are allowed to compete in the 2D model, the facultative population competitively excludes both obligate types throughout the entire domain unless it is penalized for having more metabolic capabilities. This may anticipate why many heterotrophs, including the ubiquitous SAR11, are facultatively anaerobic [61, 62].

#### A secondary chlorophyll maximum formation from O_2_-limited grazing

Phytoplankton sustainability is determined by the balance of light supply, nutrient supply, and loss rates to grazing and other mortality. Starting from the surface, phytoplankton biomass increases with depth to the deep (primary) chlorophyll maximum, where both nutrient and light supply is optimized, then decreases as light begins to limit growth, then increases again as O_2_ is depleted and grazing becomes O_2_-limited, and then decreases again because of light limitation (indicated by the chlorophyll concentration in Fig. 5e). This constitutes one hypothesis for the formation of the secondary chlorophyll maximum (SCM), and further hypothesizes that phytoplankton should experience a local minima in their loss rate there. Changes in viral lysis rates with depth may be a particularly likely hypothesis given the observation that distinct cyanophage communities exist at the SCM [63]. The model predicts simultaneous O_2_ production, O_2_ consumption, and sustained anaerobic activity at the SCM, exhibiting a “cryptic oxygen cycle” [19].

#### Formation of a secondary ${\mathrm{NO}}_2^ -$ maximum

As above, ${\mathrm{NO}}_2^ -$ accumulates in the modeled AMZ to concentrations higher than any subsistence concentration (Fig. 5f). This broad tendency arising from the stoichiometry of the redox reactions may constitute a clue for why the secondary ${\mathrm{NO}}_2^ -$ maximum forms [35]. Actual mechanisms may include time-varying concentrations of ${\mathrm{NO}}_2^ -$ [49, 50], and further differences in rate would result from significant differences in efficiencies among the anaerobic heterotrophic metabolisms.

#### Deep nitrification and carbon fixation at the AMZ periphery

Just above and below the simulated anoxic zone, significant rates of aerobic chemoautotrophic nitrification are sustained (Fig. 5g, h). This is consistent with observations [12, 14], and, along with anammox, drives deep CO_2_ fixation (Fig. 4f). In the illustrated realization, this nitrification is supported by local heterotrophic remineralization of organic matter because the one pool of utilizable (labile) organic matter remains available throughout the anoxic zone (Fig. A6d, e).

## Discussion

Theory and modeling suggest that the onset of anaerobic metabolism in aquatic environments is best characterized as a transition from competitive exclusion to stable coexistence. Even trace amounts of aerobic activity are theoretically stable if trace amounts of O_2_ are supplied, suggesting the potential for low rates of aerobic activity concurrent with anaerobic activity in anoxic environments. Adding stable coexistence to the list of explanations for co-occurring aerobic and anaerobic metabolisms (which includes particle segregation [22, 25], time-varying circulation [20, 21], and dispersal (Fig. 5h)) expands the degree to which we expect that aerobic metabolisms should be sustained “cryptically” in anoxic zones [19]. Results support the speculation that a significant amount of organic or reduced inorganic substrate may be metabolized aerobically within anoxic zones [17]. This impacts predictions of fixed nitrogen loss based on the amount of organic matter oxidized in anoxic conditions, such as in biogeochemical models that resolve organic matter fluxes.

By parameterizing diverse aerobic and anaerobic metabolisms with their underlying redox chemical reactions, the model allows for the thresholds determining the absence or presence of each metabolism to emerge dynamically as the consequence of ecological interactions. Avoiding prescribed thresholds is necessary to simulate the stable coexistence of competing metabolisms. While natural assemblages do exhibit oxygen inhibitions [18, 30], the predictions here aim to understand these inhibitions more fundamentally, i.e., to anticipate why enzymes evolved particular oxygen sensitivities over time.

The model suggests that to quantify the relative degree of aerobic and anaerobic activity, the relative supply rates of electron donor and oxygen must be known (or resolved in a physical circulation model.) For increasing organic matter input, the oxygen supply required to stay above *ϕ* also increases for constant *r* (Fig. A2). Thus, the oxygen concentration in the ocean alone is, at best, a good approximation for locations of anaerobic activity.

Results suggest that the ratio of aerobic to anaerobic respiration will generally decrease from the periphery to the core of an anoxic zone. This is consistent with the observations of foraminifera in the Peruvian AMZ [64]. The perspective here suggests that the inferred “preference” for ${\mathrm{NO}}_3^ -$ utilization by the cells in conditions where oxygen is rarely supplied constitutes the consequence, not the dictation, of the outcome of the metabolic competition among a facultative population.

Though the present 2D simulation does not capture the enhanced ${\mathrm{NO}}_2^ -$ oxidation observed in AMZs [12], we can use the framework to hypothesize the conditions that would allow it. ${\mathrm{NO}}_2^ -$ oxidation may be sustained at higher rates than ${\mathrm{NH}}_4^ +$ oxidation in the AMZ periphery where sufficient concentrations of both ${\mathrm{NO}}_2^ -$ (accumulated from ${\mathrm{NO}}_3^ -$ reduction) and O_2_ co-occur due to mixing. This is perhaps more likely deeper in the anoxic zone or at its bottom boundary where labile organic matter is depleted, inhibiting heterotrophy and ${\mathrm{NH}}_4^ +$ oxidation. Instead, the 2D model here resolves only one type of sinking organic matter that remains available beneath the simulated AMZ.

Intriguingly, the intermediate state of the chemostat model resembles some aspects of recent observations in the Bay of Bengal [33], where anammox but not denitrification was measured at intermediately low oxygen concentrations (hundreds of nM). In both model and observations, anammox is ${\mathrm{NO}}_2^ -$-limited in the intermediate state. However, unlike the observations, the model predicts that ${\mathrm{NO}}_3^ -$ reduction and denitrification are strictly coupled, which may indicate that in reality that their associated organic matter yields are not similar, that anoxic niches inside particles contributes to their spatial segregation, or that the observations reflect a non-steady state environment and so other characteristics are key [49, 50].

While the subsistence concentration R^*^ governs fitness in steady state environments, the maximum growth rate of a population is a better measure of fitness in dynamic, time-varying environments [65]. The steady state assumption is valid when the rates of activity of microbial populations are large relative to the changes in the biomass of the community, and this should characterize much of the stagnant waters where anoxic zones form. However, a pulse of fresh sinking organic matter into an anoxic zone may spur a “bloom” of microbial activity that is better characterized as a time-varying state, and the dynamics and the distinctions among the communities relevant to this state are not articulated here.

Here, we have provided a theoretical framework to quantify the distinctions between yields among microbial metabolisms. Though we focus on yields, we explained that the subsistence concentration R^*^ is the appropriate metric of fitness. Therefore, the other variables comprising R^*^ also matter for the competition. We expect large variation in uptake affinity and in loss rates due to maintenance costs, grazing, and viral lysis in the microbial community. In particular, we expect the traits of the functional types to vary consistently in different conditions, as they do for phytoplankton [65], with variation in cell size and allocation of enzyme. While we expect huge variation to characterize differences in realized phenotypes among any one metabolic strategy, we do not however anticipate systematic patterns of variation *between* metabolic strategies for these traits in ways that impact the ordering of their R^*^s. Rather, we speculate that for any given environment, an aerobe and an anaerobic analog competing for the same electron donor are dealt the same supply of elements and electrons, and have both adapted to optimize their fitness so that the only remaining distinction is the difference in the energy acquired by the respiration pathways (i.e., the free energy released by the redox reactions underlying their metabolisms). However, organisms “hard-wired” with phenotypes adapted to different environments may demonstrate a different outcome: an anaerobe adapted to environment A, for example, may outcompete an aerobe adapted to environment B if they are both subjected to environment A, and this type of interaction may characterize communities with populations supplied by dispersal from strong mixing or other transport. Of course, these differences should not matter for facultatively anaerobic populations.

A useful aspect of this modeling approach is the prediction of concentrations of active biomass associated with each metabolism. This biomass (mol N L^−1^) can be converted to cellular abundance (cells L^−1^) with an estimate of a cell quota Q (mol N cell^−1^) and compared quantitively with genes, transcripts, and other biomolecular data (e.g., [37]). The resulting insight into the relationship between community composition and function is one benefit of resolving populations explicitly in ecosystem models. For example, though substrate supply dictates that the water column anammox rate is about 30% of the denitrification rate, modeled anammox biomass is about 20x lower than denitrifying biomass in Fig. 3 because of their different substrate yields (Fig. 1). Thus, the model articulates how populations with relatively low abundances are responsible for high ambient metabolic rates and thus can be as biogeochemically significant as populations with higher abundances.

To conclude, we used redox chemistry and physiological parameterizations of resource uptake to construct a self-consistent ecosystem model that aims to deepen our understanding of microbial ecology and biogeochemistry. The theory predicts a dynamic transition from aerobic to stably coexisting aerobic and anaerobic metabolism as a function of substrate supply, and the resulting model simulates key aspects of AMZs. The approach progresses microbial ecological modeling towards resolving bulk community metabolism dynamically and systematically using underlying chemical and physical constraints, ultimately improving predictions of microbial activity in unobserved and future environments.

## Supplementary information

Supplemental Material
