# Supplementary material for: Stable aerobic and anaerobic coexistence in anoxic marine zones
Source: ISME J. 2019 Oct 17;14(1):288–301. doi: 10.1038/s41396-019-0523-8 (PMC6908664; doi:10.1038/s41396-019-0523-8)
Supplement: Supplementary file 1 — Supplemental Material [file 41396_2019_523_MOESM1_ESM.pdf]

Supplementary Information: Stable aerobic and anaerobic coexistence in anoxic marine zones

Zakem et al.

Appendix 1: Derivation of ϕ

The concentration of aerobic biomass from the steady state balance of Eqn. T4 ($\frac{d\mathrm{O}_2}{dt} = 0$) is:

$$B_O = \frac{D}{\mu_O}(\mathrm{O}_{2in} - \mathrm{O}_2^*)y_{\mathrm{O}_2} \tag{A1}$$

Thus, for aerobic biomass to exist, $\mathrm{O}_{2in} > \mathrm{O}_2^*$.

The relationship of the steady state biomasses of both the aerobic and anaerobic populations from Eqn. T3 ($\frac{dOM}{dt} = 0$) is:

$$0 = D(OM_{in} - OM_N^*) - \frac{1}{y_{OM_O}}\mu_O B_O - \frac{1}{y_{OM_N}}\mu_N B_N \tag{A2}$$

The organic matter subsistence concentration for anaerobic biomass OM_N^* is the relevant subsistence concentration for this expression, since we are working towards an expression for the coexistence of both populations, and this is the larger. (If $OM_{in} < OM_N^*$ and $OM_{in} \geq OM_O^*$), aerobic but not anaerobic biomass can accumulate.) Since $\mu = D$ at steady state in the chemostat, further simplification can be made for the chemostat, but we retain these values in order to later extend the expression to natural environments.

Plugging Eqn. A1 into Eqn. A2, rearranging gives an expression for anaerobic biomass:

$$B_N = y_{OM_N} D(OM_{in} - OM_N^*) - \frac{y_{OM_N}}{y_{OM_O}} D(\mathrm{O}_{2in} - \mathrm{O}_2^*) y_{\mathrm{O}_2} \tag{A3}$$

Thus, for anaerobic biomass to exist,

$$0 < D(OM_{in} - OM_N^*) - \frac{y_{\mathrm{O}_2}}{y_{OM_O}} D(\mathrm{O}_{2in} - \mathrm{O}_2^*) \tag{A4}$$

$$\frac{y_{\mathrm{O}_2}}{y_{OM_O}} D(\mathrm{O}_{2in} - \mathrm{O}_2^*) < D(OM_{in} - OM_N^*) \tag{A5}$$

$$\frac{y_{\mathrm{O}_2}}{y_{OM_O}} \frac{D(\mathrm{O}_{2in} - \mathrm{O}_2^*)}{D(OM_{in} - OM_N^*)} < 1 \tag{A6}$$

In the main text, we label the LHS expression as ϕ, and thus the threshold $\phi = 1$ is relevant for identifying the domain of coexistant aerobic and anaerobic biomass. We also use r to represent the ratio of oxygen to organic matter demand: $r = y_{OM_O} y_{O_2}^{-1}$ (mol O_2 utilized per mol OM utilized), and so:

$$\phi = \frac{D(\mathrm{O}_{2in} - \mathrm{O}_2^*)}{D(OM_{in} - OM_N^*)} r^{-1} \tag{A7}$$

Appendix 2: Redox-based descriptions of metabolisms

Following the methodology of Rittman and McCarty (2001), three half-reactions combine to form the catabolic and anabolic full reactions for each metabolism: 1. the oxidation of an electron donor, 2. the reduction of oxygen or nitrogen as an electron acceptor, and 3. biomass synthesis. Electron fraction parameter f partitions the electron flow towards biomass synthesis (f) vs. towards respiration for energy ($1 - f$).

We describe the N-cycling in AMZs using a minimum set of metabolisms, described below. Though natural assemblages may carry out a diversity of pathway lengths of the full denitrification reaction (Zumft 1997), we here resolve the denitrification pathway with two discrete steps, as do other modeling approaches (Penn et al. 2016; Babbin et al. 2017), because the intermediate NO_2^- is critical to AMZ biogeochemistry. We do not resolve N_2O because the small amounts formed have negligible impact on the stoichiometries of these metabolisms. We do not consider anaerobic nitrite oxidation because our understanding the process is still emerging (Babbin et al. 2017), though we provide the tools with which the traits and ecology of this (and other) metabolisms could also be examined.

Aerobic heterotrophy For the aerobic heterotroph, organic matter (OM) provides the elements and electrons for both the synthesis of biomass (B) and energy production, and oxygen serves as the electron acceptor. The three half-reactions, with notation of the factor by which each is multiplied

before summing to give the whole metabolism, are:

$$
\begin{aligned}
(1)\ & \left[\frac{1}{d_{OM}}\mathrm{C}_{c_{OM}}\mathrm{H}_{h_{OM}}\mathrm{O}_{o_{OM}}\mathrm{N}_{n_{OM}} + \frac{2c_{OM} - o_{OM} + n_{OM}}{d_{OM}}\mathrm{H_2O}\right. \\
& \quad \left. \rightarrow \quad \frac{n_{OM}}{d_{OM}}\mathrm{NH_4^+} + \frac{c_{OM} - n_{OM}}{d_{OM}}\mathrm{CO_2} + \frac{n_{OM}}{d_{OM}}\mathrm{HCO_3^-} + \mathrm{H^+} + e^-\right] \\
(1-f)\ & \left[\frac{1}{4}\mathrm{O_2} + \mathrm{H^+} + e^- \rightarrow \frac{1}{2}\mathrm{H_2O}\right] \\
(f)\ & \left[\frac{n_B}{d_B}\mathrm{NH_4^+} + \frac{c_B - n_B}{d_B}\mathrm{CO_2} + \frac{n_B}{d_B}\mathrm{HCO_3^-} + \mathrm{H^+} + e^- \rightarrow \frac{1}{d_B}\mathrm{C}_{c_B}\mathrm{H}_{h_B}\mathrm{O}_{o_B}\mathrm{N}_{n_B} + \frac{2c_B - o_B + n_B}{d_B}\mathrm{H_2O}\right]
\end{aligned}
$$

Summing the above gives the balance for the whole metabolism, here substituting organic matter $OM = \mathrm{C}_{c_{OM}}\mathrm{H}_{h_{OM}}\mathrm{O}_{o_{OM}}\mathrm{N}_{n_{OM}}$ and biomass $B_{HetO} = \mathrm{C}_{c_B}\mathrm{H}_{h_B}\mathrm{O}_{o_B}\mathrm{N}_{n_B}$, and ignoring water and lumping bicarbonate into the $\mathrm{CO_2}$ pool for brevity, as:

$$\frac{1}{d_{OM}}OM + \frac{1-f}{4}\mathrm{O_2} \rightarrow \frac{f}{d_B}B_{HetO} + \left(\frac{c_{OM}}{d_{OM}} - \frac{c_B f}{d_B}\right)\mathrm{CO_2} + \left(\frac{n_{OM}}{d_{OM}} - \frac{n_B f}{d_B}\right)\mathrm{NH_4^+} \qquad \text{(A8)}$$

where d normalizes the organic reactions to one electron. In the half-reactions for organic matter decomposition or biomass synthesis, d represents the number of electron equivalents that correspond to the oxidation states of the inorganic constituents (Rittman and McCarty 2001). For generic organic composition $\mathrm{C}_c\mathrm{H}_h\mathrm{O}_o\mathrm{N}_n$, assuming that organic N is decomposed into or assimilated from N at oxidation state -3 gives $d = 4c + h - 2o - 3n$. (Different values of d may account for the assimilation of DIN species at higher oxidation states into biomass, following Rittman and McCarty (2001)). Here, we assume a microbial biomass composition of $\mathrm{C_5H_7O_2N}$ for all functional types, in accordance with the estimate of marine heterotrophic bacterial biomass C:N of 5±1 (Zimmerman et al. 2014), giving $d_B = 20$. For the generic organic substrate, we assume an average Redfieldian composition of $OM = \mathrm{C_{6.6}H_{10.9}O_{2.6}N}$ (Anderson 1995), giving $d_{OM} = 29.1$.

The organic matter yield (or growth efficiency; mol B synthesized per mol OM consumed) relates to f as:

$$y_{OM} = \frac{d_{OM}}{d_B}f \qquad \text{(A9)}$$

and so $y_{OM} \approx f$ if the stoichiometries and redox states of the organic matter substrate and microbial biomass are similar, and $y_{OM} = 1.45f$ for our assumed Redfieldian organic substrate and microbial biomass stoichiometries.

For the aerobic heterotroph, we assign $y_{OM_{BhetO}} = 0.14$, the average marine growth efficiency in the open ocean compiled by Robinson (2008). This translates to $f = 0.096$, and gives a stoichiometry normalized to one mole of biomass as:

$$7.1OM + 47\mathrm{O}_2 \rightarrow B_{HetO} + 42\mathrm{CO}_2 + 6.1\mathrm{NH}_4^+ \tag{A10}$$

For the following anaerobic heterotrophic metabolisms, the above half-reactions are the same except for the electron acceptor half-reaction. For the anaerobic heterotrophs, we assign the same organic matter yield to all: $y_{OM_{Bhet_i}} = 0.9y_{OM_{BhetO}}$, giving $f = 0.087$, in accordance with our assumption that oxygen is a superior electron acceptor for the range of oxygen concentrations resolved for our generic organic substrate. LaRowe and Van Cappellen (2011) show that this may be so for a wide range of oxygen and DIN concentrations for the oxidation of glucose. This allows the anaerobic types to coexist in the steady state model. Further attention to time-varying states is needed to explain coexistences among anaerobic heterotrophic types if organic matter yields differ significantly.

We also note how ratio r in the equations for ϕ can be written in terms of f. Since $r = y_{OM_O} y_{O_2}^{-1}$ (mol O_2 utilized per mol OM utilized),

$$r = \frac{d_{OM}(1-f)}{4} \tag{A11}$$

Thus r increases as f decreases, and converges to $d_{OM}/4$ at low efficiencies (Fig. A1). With our assumed organic matter stoichiometry, r is about 6.6 mol O_2 per mol organic N, or about 1 mol O_2 per mol organic C, which is close to the inverse of the "respiratory quotient" for algal material of 0.9 moles of CO_2 produced per mol O_2 consumed (Robinson 2008).

Nitrate-reducing heterotrophy For NO_3^- reduction to NO_2^-, organic matter (OM) provides the elements and electrons for both the synthesis of biomass (B) and energy production, and NO_3^- serves as the electron acceptor. The full metabolism forming biomass B_{HetNO_3} is:

$$\frac{1}{d_{OM}}OM+\frac{1-f}{2}NO_3^- \rightarrow \quad \frac{f}{d_B}B_{HetNO_3}+\left(\frac{c_{OM}}{d_{OM}}-\frac{c_Bf}{d_B}\right)CO_2+\left(\frac{n_{OM}}{d_{OM}}-\frac{n_Bf}{d_B}\right)NH_4^++\frac{1-f}{2}NO_2^- \quad \text{(A12)}$$

68 With $f = 0.087$, the stoichiometry normalized to one mole of biomass is:

$$7.9OM + 105NO_3^- \rightarrow B_{HetNO3} + 47CO_2 + 6.9NH_4^+ + 105NO_2^- \quad \text{(A13)}$$

69 **Denitrifying heterotrophy** For the denitrification of NO_2^- to gaseous elemental form, organic
70 matter (OM) provides the elements and electrons for both the synthesis of biomass (B) and energy
71 production, and either NO_3^- or NO_2^- serves as the electron acceptor, which is then reduced to an
72 unspecified combination of N_2 and N_2O. Using NO_3^- as an electron acceptor (as in Fig. 2 in main
73 text), the full metabolism forming biomass B_N is:

$$\frac{1}{d_{OM}}OM+\frac{1-f}{5}NO_3^- \rightarrow \quad \frac{f}{d_B}B_N+\left(\frac{c_{OM}}{d_{OM}}-\frac{c_Bf}{d_B}\right)CO_2+\left(\frac{n_{OM}}{d_{OM}}-\frac{n_Bf}{d_B}\right)NH_4^++\frac{1-f}{10}N_2 \quad \text{(A14)}$$

74 With $f = 0.087$, the stoichiometry normalized to one mole of biomass is:

$$7.9OM + 42NO_3^- \rightarrow B_N + 47CO_2 + 6.9NH_4^+ + 21N_2 \quad \text{(A15)}$$

75 Using NO_2^- as an electron acceptor (all but Fig. 2 in main text), the full metabolism forming biomass
76 B_{HetNO_2} is:

$$\frac{1}{d_{OM}}OM+\frac{1-f}{3}NO_2^- \rightarrow \quad \frac{f}{d_B}B_{HetNO_2}+\left(\frac{c_{OM}}{d_{OM}}-\frac{c_Bf}{d_B}\right)CO_2+\left(\frac{n_{OM}}{d_{OM}}-\frac{n_Bf}{d_B}\right)NH_4^++\frac{1-f}{6}N_2 \quad \text{(A16)}$$

With $f = 0.087$, the stoichiometry normalized to one mole of biomass is:

$$7.9OM + 70\mathrm{NO}_2^- \rightarrow B_{HetNO_2} + 47\mathrm{CO}_2 + 6.9\mathrm{NH}_4^+ + 35\mathrm{N}_2 \tag{A17}$$

DNRA For organisms carrying out dissimilatory nitrate (or nitrite) reduction to ammonium (DNRA), organic matter (OM) provides the elements and electrons for both the synthesis of biomass (B) and energy production, and either NO_3^- or NO_2^- serves as the electron acceptor, which is reduced to NH_4^+. When using NO_3^- as an electron acceptor, the full metabolism can be approximated as:

$$\frac{1}{d_{OM}}OM + \frac{1-f}{8}\mathrm{NO}_3^- \rightarrow \quad \frac{f}{d_B}B_{HetDNRA_{NO_3}} + \left(\frac{c_{OM}}{d_{OM}} - \frac{c_B f}{d_B}\right)\mathrm{CO}_2 + \left(\frac{n_{OM}}{d_{OM}} - \frac{n_B f}{d_B} + \frac{1-f}{8}\right)\mathrm{NH}_4^+ \tag{A18}$$

For the $f = 0.087$ assumed for all anaerobic heterotrophs, the stoichiometry normalized to one mole of biomass is:

$$7.9OM + 26\mathrm{NO}_3^- \rightarrow B_{HetDNRA_{NO_3}} + 47\mathrm{CO}_2 + 33\mathrm{NH}_4^+ \tag{A19}$$

When using NO_2^- as an electron acceptor for DNRA, the full metabolism can be approximated as:

$$\frac{1}{d_{OM}}OM + \frac{1-f}{6}\mathrm{NO}_2^- \rightarrow \quad \frac{f}{d_B}B_{HetDNRA_{NO_2}} + \left(\frac{c_{OM}}{d_{OM}} - \frac{c_B f}{d_B}\right)\mathrm{CO}_2 + \left(\frac{n_{OM}}{d_{OM}} - \frac{n_B f}{d_B} + \frac{1-f}{6}\right)\mathrm{NH}_4^+ \tag{A20}$$

and with $f = 0.087$, the stoichiometry normalized to one mole of biomass is:

$$7.9OM + 35\mathrm{NO}_2^- \rightarrow B_{HetDNRA_{NO_2}} + 47\mathrm{CO}_2 + 42\mathrm{NH}_4^+ \tag{A21}$$

Solutions including both of these metabolisms as metabolic functional types in the chemostat are plotted in Fig. A3 and discussed in Appendix 3.

Chemoautotrophic aerobic NH_4^+ oxidation For the NH_4^+ oxidizer (here considering NH_4^+ and NH_3 interchangeably), as in Zakem et al. (2018), the three half-reactions, for generic biomass $C_cH_hO_oN_n$, and their electron-partitioning coefficients, are:

$$
\begin{aligned}
(1)\quad & \left[\frac{1}{6}NH_4^+ + \frac{1}{3}H_2O \rightarrow \frac{1}{6}NO_2^- + \frac{4}{3}H^+ + e^-\right] \\
(1-f)\quad & \left[\frac{1}{4}O_2 + H^+ + e^- \rightarrow \frac{1}{2}H_2O\right] \\
(f)\quad & \left[\frac{n}{d}NH_4^+ + \frac{c-n}{d}CO_2 + \frac{n}{d}HCO_3^- + H^+ + e^- \rightarrow \frac{1}{d}C_cH_hO_oN_n + \frac{2c-o+n}{d}H_2O\right]
\end{aligned}
$$

89 The sum gives the full metabolism for NH_4^+-oxidizing biomass B_{AOO} , as a function of f:

$$\left(\frac{1}{6} + \frac{f}{d}\right) NH_4^+ + \frac{cf}{d}CO_2 + \frac{1-f}{4}O_2 \rightarrow \frac{f}{d}B_{AOO} + \frac{1}{6}NO_2^- \qquad \text{(A22)}$$

90 For $f = 0.03$, the stoichiometry normalized to one mole of biomass is:

$$112NH_4^+ + 5CO_2 + 162O_2 \rightarrow B_{AOO} + 111NO_2^- \qquad \text{(A23)}$$

Chemoautotrophic aerobic NO_2^- oxidation For the NO_2^- oxidizer, as in Zakem et al. (2018), the three half-reactions are:

$$
\begin{aligned}
(1)\quad & \left[\frac{1}{2}NO_2^- + \frac{1}{2}H_2O \rightarrow \frac{1}{2}NO_3^- + H^+ + e^-\right] \\
(1-f)\quad & \left[\frac{1}{4}O_2 + H^+ + e^- \rightarrow \frac{1}{2}H_2O\right] \\
(f)\quad & \left[\frac{n}{d}NH_4^+ + \frac{c-n}{d}CO_2 + \frac{n}{d}HCO_3^- + H^+ + e^- \rightarrow \frac{1}{d}C_cH_hO_oN_n + \frac{2c-o+n}{d}H_2O\right]
\end{aligned}
$$

91 which when summed gives the full metabolism NO_2^--oxidizing biomass B_{NOO} as:

$$\frac{1}{2}NO_2^- + \frac{f}{d}NH_4^+ + \frac{cf}{d}CO_2 + \frac{1-f}{4}O_2 \rightarrow \frac{f}{d}B_{NOO} + \frac{1}{2}NO_3^- \qquad \text{(A24)}$$

where the requirement of one mole of NH_4^+ per mole NOO biomass is effectively negligible in all model simulations. For $f = 0.03$, the stoichiometry normalized to one mole of biomass is:

$$334\mathrm{NO_2^-} + 5\mathrm{CO_2} + 162\mathrm{O_2} \rightarrow B_{NOO} + 334\mathrm{NO_3^-} \qquad \text{(A25)}$$

Chemoautotrophic anammox For chemoautotrophic anaerobic ammonium oxidation (anammox), NH_4^+ oxidation to elemental N provides electrons for energy that fuels cell synthesis (here considering NH_4^+ and NH_3 interchangeably), and NO_2^- serves as the electron acceptor, forming elemental N_2. Anammox is observed to also excrete NO_3^- (Strous et al. 1998). Thus, the whole metabolism may be characterized by considering two electron donor half reactions for NH_4^+: oxidation to NO_3^- and oxidation to N_2. With this simplified form, the following four half-reactions can represent the anammox metabolism, with x dictating the weighting of the electron donor reaction:

$$\begin{aligned}
(x) \quad & \left[\frac{1}{3}\mathrm{NH_4^+} \rightarrow \frac{1}{6}\mathrm{N_2} + \frac{4}{3}\mathrm{H^+} + e^-\right] \\
(1-x) \quad & \left[\frac{1}{8}\mathrm{NH_4^+} \rightarrow \frac{1}{8}\mathrm{NO_3^-} + \frac{1}{2}\mathrm{H^+} + e^-\right] \\
(1-f) \quad & \left[\frac{1}{3}\mathrm{NO_2^-} + \frac{4}{3}\mathrm{H^+} + e^- \rightarrow \frac{1}{6}\mathrm{N_2} + \frac{2}{3}\mathrm{H_2O}\right] \\
(f) \quad & \left[\frac{n}{d}\mathrm{NH_4^+} + \frac{c-n}{d}\mathrm{CO_2} + \frac{n}{d}\mathrm{HCO_3^-} + \mathrm{H^+} + e^- \rightarrow \frac{1}{d}\mathrm{C}_c\mathrm{H}_h\mathrm{O}_o\mathrm{N}_n + \frac{2c-o+n}{d}\mathrm{H_2O}\right]
\end{aligned}$$

This gives the full metabolism forming anammox biomass B_{anx} as:

$$\left(\frac{x}{3} + \frac{1-x}{8} + \frac{f}{d}\right)\mathrm{NH_4^+} + \frac{1-f}{3}\mathrm{NO_2^-} + \frac{cf}{d}\mathrm{CO_2} \rightarrow \frac{f}{d}B_{anx} + \frac{1-x}{8}\mathrm{NO_3^-} + \frac{x+1-f}{6}\mathrm{N_2} \qquad \text{(A26)}$$

To estimate an appropriate value for x, we calibrate this with the stoichiometry of anammox in wastewater reported by Strous et al. (1998). Normalized to one mole of N-based biomass for comparison, this published stoichiometry is:

$$101\mathrm{NH_4^+} + 133\mathrm{NO_2^-} + 6.6\mathrm{CO_2} \rightarrow B_{anx} + 26.2\mathrm{NO_3^-} + 103\mathrm{N_2} \qquad \text{(A27)}$$

This shows that anammox produces NO_3^- to N_2 at a ratio of approximately 1:4. From this, we can solve for $x \approx 0.5$. With $x = 0.5$, we find that the stoichiometry of Strous et al. (1998) is approximated when the value of f is 0.05:

$$93NH_4^+ + 127NO_2^- + 5CO_2 \rightarrow B_{anx} + 25NO_3^- + 97N_2 \tag{A28}$$

However, for all three chemoautotrophic metabolic functional types, we assume $f = 0.03$ in the illustrated solutions. This is because Zakem et al. (2018) used theoretical and empirical analysis to estimate that the electron fraction f for both steps of aerobic nitrification was significantly lower in marine environments ($f \approx 0.03$) than in wastewater ($f \approx 0.1$). Furthermore, the published stoichiometry for anammox in wastewater suggests value of f lower than those of aerobic ammonia oxidation in wastewater ($f \approx 0.05$) (Strous et al. 1998; Rittman and McCarty 2001). Thus if the degree to which anammox efficiency is lower than aerobic nitrifier efficiency in the ocean can be approximated by the ratio of anammox to nitrifier efficiency in wastewater (0.05:0.1), a value of $f \approx 0.03/2 \approx 0.015$ may be a justifiable estimate for anammox efficiency in the ocean. However, given the uncertainty, and to most robustly test the competition between them, we assigned the same value of $f = 0.03$ to anammox as well as to the nitrifiers. With $f = 0.03$ and $x = 0.5$, the anammox stoichiometry is:

$$154NH_4^+ + 216NO_2^- + 5CO_2 \rightarrow B_{anx} + 42NO_3^- + 163N_2 \tag{A29}$$

Appendix 3: Detail for the multiple redox-based metabolisms in the virtual chemostat

Equations Equations for the multiple metabolisms in the chemostat are similar in form to those in Table 1 in the main text. Organic matter, O_2, and NO_3^- are supplied to chemostat. The biomass B_i (µM N) of each metabolic functional type i, organic matter (µM N), and all nutrients (µM) are resolved by solving their rates of change with time defined by incoming nutrient supply, nutrient uptake, growth rate, excretion of waste respiration products, and the chemostat dilution rate D as:

$$\frac{dB_i}{dt} = B_i(\mu_i - D) \tag{A30}$$

$$\frac{dOM}{dt} = D(OM_{in} - OM) - \sum_i \frac{1}{y_{OM_i}} \mu_i B_i \tag{A31}$$

$$\frac{d[\mathrm{NH_4^+}]}{dt} = \sum_i e_{NH_{4i}} \mu_i B_i - \sum_i \frac{1}{y_{NH_{4i}}} \mu_i B_i - D[\mathrm{NH_4^+}] \tag{A32}$$

$$\frac{d[\mathrm{NO_2^-}]}{dt} = \sum_i e_{NO_{2i}} \mu_i B_i - \sum_i \frac{1}{y_{NO_{2i}}} \mu_i B_i - D[\mathrm{NO_2^-}] \tag{A33}$$

$$\frac{d[\mathrm{NO_3^-}]}{dt} = \sum_i e_{NO_{3i}} \mu_i B_i - \sum_i \frac{1}{y_{NO_{3i}}} \mu_i B_i - D[\mathrm{NO_3^-}] \tag{A34}$$

$$\frac{d[\mathrm{O_2}]}{dt} = D([\mathrm{O_{2in}}] - [\mathrm{O_2}]) - \sum_i \frac{1}{y_{O_{2i}}} \mu_i B_i \tag{A35}$$

where yields y and excretion ratios e are listed in Table A1. Each growth rate μ is calculated according to Eqn. 1 in the main text using the yields and uptake kinetic parameters in Tables A1 and A2.

Model ensemble To consider uncertainty in the parameterizations, we computed an ensemble of 1000 model simulations for which parameters were randomly sampled from a distribution as follows. The efficiency of the heterotrophic metabolisms overall was varied, the degree to which anaerobic heterotrophy was less efficient than aerobic heterotrophy was varied, and the efficiencies

of the three chemoautotrophic metabolisms were independently varied. Since we expect variation in these yields, but not the underlying energetic constraints, we vary the overall heterotrophic growth efficiency but retain the lower efficiency of the anaerobic heterotrophs. The aerobic heterotrophic yield $y_{OM_{BhetO}}$ was varied over a linear range from 0.1 to 0.3 (mol biomass synthesized per mol OM utilized). The anaerobic heterotrophic yields were less this yield by a factor of 1% to 50%, varying linearly over this range. The three chemoautotrophic yields were independently varied by varying their electron fraction f over a linear range from $f = 0.02$ to $f = 0.04$. This allowed the potential for cases in which the anammox NH_4^+ yield was higher than that of the aerobic NH_4^+ oxidizer, potentially resulting in sustainable anammox in the oxygenated state, but this case did not occur in any of the 1000 solutions.

An argument for low efficiency of DNRA The model solutions without DNRA are illlustrated in the main text (Fig. 3), and with DNRA in Fig. A3. When the DNRA functional type is included, NH_4^+ but not NO_2^- accumulates to micromolar concentrations in the anoxic state (Fig. A3). This is consistent with some observations in the South Pacific oxygen minimum zone of Kalvelage et al. (2013): at the few locations where measured DNRA rates were significantly high (10–1000 nM d^{-1}), NH_4^+ concentrations were also significantly high (1–5 μM), and NO_2^- concentrations were low. Since DNRA rates were low throughout the rest of the dataset (less than about 1 nM d^{-1}), and since in general AMZs show characteristic accumulation of NO_2^- and not NH_4^+, with an assessment of the literature we agree with the speculation that DNRA operates sporadically in AMZs (Lam et al. 2009; Jensen et al. 2011; Füssel et al. 2012; Kalvelage et al. 2013). The specific mechanism sustaining DNRA remains unclear, but this does hypothesize that DNRA may be associated with a lower efficiency than other anaerobic metabolisms. In the steady state solutions, DNRA is excluded if its efficiency is lower, and so we further speculate that favorable DNRA may require a time-varying environment to avoid competitive exclusion as an ‘r-selected’ vs. a ’k-selected’ metabolism.

Table A1: Parameters for metabolic functional type yields y (inverse values listed) and excretions e from redox-based descriptions (see chemical equations in Appendix B). Units for y^{-1} and e are mol substrate per mol biomass N synthesized.

Type	Parameter	Symbol	Value (mol/mol)	Source
**Two metabolisms (Fig. 2 case study):**				
B_O	Organic matter demand	$y^{-1}_{OM_{B_O}}$	7.1	Robinson (2008)
	Oxygen demand	$y^{-1}_{O_2}$	47	Eqns. A8 and A9
B_N	Organic matter demand	$y^{-1}_{OM_{B_N}}$	7.9	$y_{OM_N} = 0.9 \cdot y_{OM_O}$
	DIN demand	y^{-1}_N	42	Eqns. A9 and A14
**Multiple metabolisms (Fig. 3, for chemostat and 2D model):**				
B_{HetO}	Organic matter demand	$y^{-1}_{OM_{BhetO}}$	7.1	Robinson (2008)
	Oxygen demand	$y^{-1}_{O_{2BhetO}}$	47	Eqns. A8 and A9
	Ammonium excretion	$e_{NH_{4BhetO}}$	6.1	Eqns. A8 and A9
B_{HetNO_3}	Organic matter demand	$y^{-1}_{OM_{BhetNO_3}}$	7.9	$y_{OM_{Bhet_i}} = 0.9 \cdot y_{OM_{BhetO}}$
	Nitrate demand	$y^{-1}_{NO_{3BhetNO_3}}$	105	Eqns. A9 and A12
	Ammonium excretion	$e_{NH_{4BhetNO_3}}$	6.9	Eqns. A9 and A12
	Nitrite excretion	$e_{NO_{2BhetNO_3}}$	105	Eqns. A9 and A12
B_{HetNO_2}	Organic matter demand	$y^{-1}_{OM_{BhetNO_2}}$	7.9	$y_{OM_{Bhet_i}} = 0.9 \cdot y_{OM_{BhetO}}$
	Nitrite demand	$y^{-1}_{NO_{2BhetNO_2}}$	70	Eqns. A9 and A16
	Ammonium excretion	$e_{NH_{4BhetNO_2}}$	6.9	Eqns. A9 and A16
	N_2 (or N_2O) excretion	$e_{N_{2BhetNO_2}}$	35	Eqns. A9 and A16
B_{AOO}	Ammonium demand	$y^{-1}_{NH_{4B_{AOO}}}$	112	$f = 0.03$; Zakem et al. (2018)
	Oxygen demand	$y^{-1}_{O_{2B_{AOO}}}$	162	Eqn. A22
	Nitrite excretion	$e_{NO_{2B_{AOO}}}$	111	Eqn. A22
B_{NOO}	Nitrite demand	$y^{-1}_{NO_{2B_{NOO}}}$	334	$f = 0.03$; Zakem et al. (2018)
	Oxygen demand	$y^{-1}_{O_{2B_{NOO}}}$	162	Eqn. A24
	Nitrate excretion	$e_{NO_{3B_{NOO}}}$	334	Eqn. A24
B_{anx}	Ammonium demand	$y^{-1}_{NH_{4B_{anx}}}$	154	$f = 0.03$
	Nitrite demand	$y^{-1}_{NO_{2B_{anx}}}$	216	Eqn. A26
	Nitrate excretion	$e_{NO_{3B_{anx}}}$	42	Eqn. A26, $x = 0.5$
	N_2 excretion	$e_{N_{2B_{anx}}}$	163	Eqn. A26, $x = 0.5$
**DNRA (included in Fig. A3 only):**				
$B_{HetDNRA_{NO_3}}$	Organic matter demand	$y^{-1}_{OM_{BhetDNRA-NO_3}}$	7.9	$y_{OM_{Bhet_i}} = 0.9 \cdot y_{OM_{BhetO}}$
	Nitrate demand	$y^{-1}_{NO_{3BhetDNRA-NO_3}}$	26	Eqns. A9 and A18
	Ammonium excretion	$e_{NH_{4BhetDNRA-NO_3}}$	33	Eqns. A9 and A18
$B_{HetDNRA_{NO_2}}$	Organic matter demand	$y^{-1}_{OM_{BhetDNRA-NO_2}}$	7.9	$y_{OM_{Bhet_i}} = 0.9 \cdot y_{OM_{BhetO}}$
	Nitrite demand	$y^{-1}_{NO_{2BhetDNRA-NO_2}}$	35	Eqns. A9 and A20
	Ammonium excretion	$e_{NH_{4BhetDNRA-NO_2}}$	42	Eqns. A9 and A20

Table A2: Uptake kinetic parameters for metabolic functional types.

Substrate	Parameter	Symbol	Value (mol/mol B)	Units	Source
OM	Maximum uptake rate	V_{maxOM}	1 (0.5 in 2D)	mol OM mol B^{-1} d^{-1}	*
	Half-saturation conc.	K_{OM}	0.1 (0.01 in 2D)	µM OM	
DIN	Maximum uptake rate	V_{maxN}	50.8	mol N mol B^{-1} d^{-1}	Martens-Habbena et al. (2009); Zakem et al. (2018)
	Half-saturation conc.	K_N	133	nM N	Martens-Habbena et al. (2009)
O_2	Cell radius	r	0.25	µm	
	Cell C quota**	q	18.3	fmol C µm^{-3}	Bratbak and Dundas (1984)
	Diffusion coefficient for O_2 in seawater	$\mathcal{D}$	$1.5 \cdot 10^{-5}$	cm^2 s^{-1}	Unisense Seawater and Gases

*The kinetic parameters for organic matter uptake rate were estimated from the average marine bacterial growth efficiency of order 0.1 d^{-1} with the estimate of the average bulk marine bacterial growth rate of order 0.1 d^{-1}. A maximum uptake rate of $V_{maxOM} \approx 1$ allows this growth rate when organic matter is abundant. The chosen half-saturation constant is arbitrarily chosen to allow for depletion of the average (non-recalcitrant) organic matter pool.
**A C:N of 5 for microbial biomass is used to express yields in units of biomass N.

Appendix 4: 2D model detail

Flow field

Governing Equations A two-dimensional, basin-wide closed flow field is developed (in the $x - z$ plane with no gradients in y) using the governing momentum equations:

$$\frac{\partial u}{\partial t} = -u\frac{\partial u}{\partial x} - w\frac{\partial u}{\partial z} - \frac{1}{\rho_0}\frac{\partial p}{\partial x} + fv + \bigtriangledown \cdot \kappa \bigtriangledown u \tag{A36}$$

$$\frac{\partial v}{\partial t} = -u\frac{\partial v}{\partial x} - w\frac{\partial v}{\partial z} - fu + \bigtriangledown \cdot \kappa \bigtriangledown v \tag{A37}$$

A wind stress in the y direction, $\tau = (0, \tau^y)$, is imposed by the surface boundary condition $\kappa\frac{\partial v}{\partial z} = \tau^y/\rho$, where ρ is the density of seawater. With horizontal u computed, a non-divergent 2D circulation field can be computed from continuity as:

$$\frac{\partial u}{\partial x} + \frac{\partial w}{\partial z} = 0 \tag{A38}$$

and integrating downwards (or upwards) to solve for the vertical velocity field w, with $w = 0$ as the top (or bottom) boundary condition.

The Pressure Field The wind-driven Ekman transport in the x-direction sets up an overturning circulation through the horizontal pressure gradient. Assuming the pressure to be hydrostatic, the horizontal pressure gradient can be decomposed into the baroclinic pressure gradient, calculated from density anomalies, and the surface pressure gradient, arising from deviations in sea surface height. In this 2D circulation model, temperature and salinity are not resolved, and density anomalies are set to zero throughout the domain. Thus the horizontal pressure gradient is depth-independent

and consists of only the surface pressure gradient, as:

$$\frac{\partial p}{\partial x} = \frac{\partial p_{surf}}{\partial x} \tag{A39}$$

Using the rigid lid approximation as a constraint, the total flux in and out of each water column must sum to zero, and can be expressed in discretized form as:

$$\sum_{j=1}^{nj} (u_{j,i+1} - u_{j,i}) dz_j = 0, \tag{A40}$$

where the grid cell index i refers to the x-direction, j to the z-direction, and dz is the height of the grid cell. Substituting Eqn. A39 into Eqn. A36 (momentum in the x-direction) and then inserting this into Eqn. A40 allows for solving the horizontal surface pressure derivative. For a boundary condition, $\frac{\partial p}{\partial x} = 0$ was imposed on the left (or right) boundary, and the pressure gradient was solved for at the face of each column by integrating from left to right (or right to left), which resulted in an analogous boundary pressure gradient of zero at the far boundary. The u velocity was then calculated with the newly updated pressure gradient at each time step, and checked for consistency with Eqn. A40.

Wind Stress Forcing The y-component of the wind stress was modeled to simulate the climatological mean from Hellerman and Rosenstein (1983) over the Pacific Basin at 10°S latitude (Fig. A4) as:

$$\tau_y(x) = 0.0125(\sin(\frac{\pi x}{0.8L} - \frac{\pi}{2}) + 1)(-\tanh(\frac{\pi x}{0.1L} - 9\pi) + 1) \tag{A41}$$

where L is the length of the domain (10,000 km).

Mixing A mixed layer was imposed by varying the vertical diffusion coefficient κ_Z with depth, from a maximum κ_{Zmax} at the surface to a minimum κ_{Zmin} with a length scale of z_{ML}. The fixed (no flux) boundary conditions result in some accumulation of POM at the bottom of the 2000 m

domain, conceptually representing a sediment layer. To diffuse this sediment layer, vertical mixing was increased within a bottom boundary mixed layer of depth scale 100 m. κ_Z (m^2 s^{-1}) is thus calculated at cell faces as:

$$\kappa_Z = \kappa_{Zmax} e^{-\frac{z}{z_{mld}}} + \kappa_{Zmin} + \kappa_{Zmax} e^{-\frac{z-H}{100}} \tag{A42}$$

where z is in meters and H is the height of the domain (2000 m). A constant value of horizontal diffusion κ_X was prescribed to account for mixing by subgrid-scale processes.

Numerical solution The momentum equations were solved to calculate the flow field with 10 m vertical resolution and and 100 km horizontal resolution over a domain 2000 m in height and 10,000 km in width. The choice to resolve the time step explicitly led to the need to resolve gravity waves, and so a 10^{-3} day time step was necessary. Equations were integrated forward in time using the 4th order Runge-Kutta method. Advection was carried out using the QUICK advection scheme, consisting of a linear interpolation between points weighted by an upstream 2nd order curvature, resulting in 3rd order accuracy. Fluxes were calculated at the faces of each grid cell, and concentrations at the centers. The resulting u and w fields used for the biogeochemistry model were saved after 100 years of spin up.

Biogeochemistry

The idealized AMZ biogeochemical model includes 17 state variables (11 populations and 6 nutrients): the biomasses of the microbial metabolic functional type populations from the chemostat model (B_{HetO}, B_{AOO}, B_{NOO}, B_{HetNO_3}, B_{HetNO_2}, and B_{anx}), two phytoplankton populations (smaller P_S and larger P_L), three zooplankton grazer populations (microzooplankton microbial grazer Z_B, and one each preying on the phytoplankton populations, Z_{P_S} and Z_{P_L}), dissolved organic matter (DOM), sinking particulate organic matter (POM), three inorganic species of DIN (NH_4^+, NO_2^-, and NO_3^-), and oxygen (O_2). All are resolved in concentrations of nitrogen except for O_2.

Total nitrogen (the sum of all nutrients, organic matter, and biomasses) is conserved. The DIN transformed to gaseous form (as N_2O or N_2) is balanced by immediately redistributing its sum evenly over the domain as nitrate, which simulates a distant source of nitrogen fixation. Oxygen fluxes across the air-sea interface according to transfer coefficient of κ_g over equilibration depth h_g according to a saturation concentration O_{2sat}. See Table A3 for parameter values.

Each tracer C is advected and diffused by the two-dimensional velocity field $\mathbf{u} = (u, w)$ and diffusion coefficients $\boldsymbol{\kappa}$ as:

$$\frac{\partial C}{\partial t} = -\nabla \cdot (\mathbf{u}C) + \nabla \cdot (\boldsymbol{\kappa}\nabla C) + S_C \tag{A43}$$

where S_C are additional sources and sinks. Growth rate μ_i for each microbial population is calculated with Eqn. 1, using the yields and uptake kinetic parameters in Tables A1 and A2, and modified by temperature by γ_T (Eqn. A60; Table A3). For each of the 17 tracers, the sources and sinks are as follows:

$$S_{B_i} = B_i(\mu_i - m_{lin} - m_q B_i - g Z_B) \tag{A44}$$

$$S_{P_i} = P_i(\mu_i - m_{lin} - m_q P_i - g Z_{P_i} \frac{\mathrm{O_2}}{\mathrm{O_2} + K_{\mathrm{O_2}}}) \tag{A45}$$

$$S_{Z_B} = Z_B(\zeta g \sum_i B_i - m_Z Z_B) \tag{A46}$$

$$S_{Z_{P_i}} = Z_{P_i}(\zeta g P_i \frac{\mathrm{O_2}}{\mathrm{O_2} + K_{\mathrm{O_2}}} - m_Z Z_{P_i}) \tag{A47}$$

$$S_{POM} = f_{mort}\left[m_{lin}\sum_i B_i + m_{lin}\sum_i P_i + m_q \sum_i B_i^2 + m_q \sum_i P_i^2 + m_Z \sum_i Z_i^2\right] - \sum_i \frac{1}{y_{OM_i}}\mu_i B_i \frac{POM}{POM + DOM} - \frac{\partial(w_s POM)}{\partial z} \tag{A48}$$

$$S_{DOM} = (1 - f_{mort})\left[m_{lin}\sum_i B_i + m_{lin}\sum_i P_i + m_q \sum_i B_i^2 + m_q \sum_i P_i^2 + m_Z \sum_i Z_i^2\right] - \sum_i \frac{1}{y_{OM_i}}\mu_i B_i \frac{DOM}{POM + DOM} \tag{A49}$$

$$S_{\mathrm{NH_4^+}} = \sum_i e_{NH_{4i}}\mu_i B_i - \sum_i \frac{1}{y_{NH_{4i}}}\mu_i B_i - \sum_i \mu_i P_i \frac{\mathrm{NH_4^+}}{\mathrm{DIN}} + (1-\zeta)g\left[\sum_i B_i Z_B + \sum_i P_i Z_{P_i}\frac{\mathrm{O_2}}{\mathrm{O_2} + K_{\mathrm{O_2}}}\right] \tag{A50}$$

$$S_{\mathrm{NO_2^-}} = \sum_i e_{NO_{2i}}\mu_i B_i - \sum_i \frac{1}{y_{NO_{2i}}}\mu_i B_i - \sum_i \mu_i P_i \frac{\mathrm{NO_2^-}}{\mathrm{DIN}} \tag{A51}$$

$$S_{\mathrm{NO_3^-}} = \sum_i e_{NO_{3i}}\mu_i B_i - \sum_i \frac{1}{y_{NO_{3i}}}\mu_i B_i - \sum_i \mu_i P_i \frac{\mathrm{NO_3^-}}{\mathrm{DIN}} + \frac{\iint (\sum_i e_{N_{2i}}\mu_i B_i)\,dxdz}{\iint dxdz} \tag{A52}$$

$$S_{\mathrm{O_2}} = \frac{\kappa_g}{h_g}(\mathrm{O}_{2sat} - \mathrm{O_2})^* + R_{\mathrm{O_2}P}\sum_i \mu_i P_i - \sum_i \frac{1}{y_{\mathrm{O}_{2i}}}\mu_i B_i - f(\mathrm{O_2}, Z_B) - R_{\mathrm{O_2}Z}(1-\zeta)g\sum_i P_i Z_{P_i}\frac{\mathrm{O_2}}{\mathrm{O_2} + K_{\mathrm{O_2}}} \tag{A53}$$

*over equilibration depth h_g

Phytoplankton Two oxygenic phytoplankton types are resolved: one representing a small, high-affinity $Prochlorococcus$-like population with a lower maximum growth rate and higher nutrient affinity, and one representing a larger, faster growing type with lower affinity. Following Dutkiewicz et al. (2015), light absorption for the smaller type is higher than that of the larger type, and so the smaller type is more fit at lower light levels deeper in the water column. Both types produce oxygen in relation to their growth with a Redfieldian O_2:N ratio (7.3:1). Phytoplankton grow as a function of a maximum growth rate μ_{max} (d^{-1}), with type-specific limitation by nutrients (γ_{N_i}), type-specific limitation by light (γ_{I_i}), and modification by temperature (γ_T) as:

$$\mu_{P_i} = \mu_{maxP_i}\gamma_{N_i}\gamma_{I_i}\gamma_T \tag{A54}$$

Nutrient limitation is a function of the total concentration of all species of DIN:

$$\gamma_{N_i} = \min\left[1, \frac{\mathrm{NH}_4^+}{\mathrm{NH}_4^+ + K_{\mathrm{NH}_4 P_i}} + \frac{\mathrm{NO}_2^-}{\mathrm{NO}_2^- + K_{\mathrm{NO}_x P_i}} + \frac{\mathrm{NO}_3^-}{\mathrm{NO}_3^- + K_{\mathrm{NO}_x P_i}}\right] \tag{A55}$$

The uptake of each DIN species by each phytoplankton type is weighted by the concentration of each substrate. The inhibition of NO_2^- and NO_3^- assimilation in the presence of NH_4^+ had a negligible effect on the solutions and so was not included. Values for the maximum growth rate and the half-saturation constants were computed as functions of cell size following data-based allometric relationships in Litchman et al. (2007) as in Ward et al. (2012). The effective half-saturation constants for DIN uptake with respect to μ_{max} were calculated with respect to maximum uptake rate V_{max} and minimum cell quota Q_{min} from the relationships in Litchman et al. (2007), following Verdy et al. (2009) and Ward et al. (2012) (Table A3).

Light limitation was parameterized using an exponential form as a function of an instantaneous photosynthetic rate and the Chl *a* to Carbon ratio θ, following Geider et al. (1997) and Hickman et al. (2010):

$$\gamma_{I_i} = 1 - \exp\left(\frac{-\Gamma_i\theta_i}{\mu_{maxP_i}\gamma_{N_i}\gamma_T}\right) \tag{A56}$$

Photosynthetic rate Γ_i for each type was computed as a function of photosynthetically active radiation $I(z)$, the maximum quantum yield of carbon fixation ϕ (mol C mol^{-1} photons), and the absorption of light by phytoplankton $a_{P_i}^{\mathrm{chl}}$ (m^2 (mgChl)$^{-1}$) representing a mean value over all wavelengths, as:

$$\Gamma_i = \phi a_{P_i}^{\mathrm{chl}} I(z) \tag{A57}$$

The Chl:C (θ) varies with photoacclimation, and is computed using a steady-state solution (Geider et al. 1997) with maximum ratio θ_{max} as:

$$\theta_i = \frac{\theta_{max}}{1 + \frac{\Gamma_i \theta_{max}}{2(\mu_{max P_i} \gamma_{N_i} \gamma_T)}} \tag{A58}$$

Grazing Three grazer populations consume oxygen, contributing to the formation of the AMZ. One population represents microzooplankton bacteriovores and consumes all of the non-photosynthetic microbial functional types. A second consumes the small phytoplankton type, and a third consumes the large phytoplankton type. Each zooplankton population grows as a linear function of its prey biomass with grazing coefficient g and growth efficiency ζ (Armstrong 1994). NH_4^+ is excreted as a waste product in proportion to $(1 - \zeta)$. Quadratic mortality rate m_Z represents predation by higher trophic levels.

Since the diffusive oxygen limitation assumed for the aerobic microbes should not apply to these larger organisms, further parameterization of oxygen limitations to zooplankton are required. For the population consuming the non-photosynthetic community (Z_B), we developed a parameterization of zooplankton oxygen consumption that implicitly simulates bacteriovore migration in and out of AMZs (Escribano et al. 2009; Wishner et al. 2013; Bianchi et al. 2014). Oxygen demand by the zooplankton at any given location is spread vertically above and below that location, and weighted by the oxygen concentration at that location, with zero weight if O_2 is below a critical oxygen concentration for zooplankton (here, 10 µM; see attached code). This mimicks zooplankton ability to breathe above or below the anoxic area, and swim into the area for grazing Escribano et al. (2009).

This loss is termed $f(\mathrm{O}_2, Z_B)$ in the differential equation for oxygen for the 2D model (Eqn. A53).

The two grazing types that consume phytoplankton are not allowed this capability, from the perspective that these grazers are adapted to oxygenated surface conditions, and are inhibited by oxygen using a saturating form in which their rate of grazing is halved at K_{O_2}. This oxygen inhibition of grazers consuming phytoplankton is a necessary (but not sufficient) condition for the formation of the secondary chlorophyll maximum in the model.

Light Light energy I decreases with depth according to the attenuation coefficients for water k_w:

$$I(z) = I_{in} e^{(-zk_w)} \tag{A59}$$

Temperature All microbial growth, grazing, and mortality rates are represented as a function of temperature (non-dimensional γ_T) using a formulation that follows the Arrhenius equation (Dutkiewicz et al. 2015) as:

$$\gamma_T = \tau \exp(A_E(\frac{1}{T} - \frac{1}{T_0})) \tag{A60}$$

where T is the ambient temperature (K), T_0 is a reference temperature, A_E regulates the temperature modification, and τ normalizes the maximum value. The model assumes a constant temperature profile, an average of the 10°S Pacific Ocean transect from the WOA 2013 climatology. This temperature dependency increases microbial rates by a factor of three from the deep to the surface, but does not impact solutions qualitatively.

Numerical solution The biogeochemical model was run 'offline' using the above flow field with 10 m vertical resolution and 100 km horizontal resolution. Particulate organic matter was additionally advected by constant sinking velocity w_s. The air-sea flux of oxygen is resolved as an open boundary with a fixed equilibrium concentration as described above. Solutions were integrated until an equilibrium state was reached.

Table A3: Additional parameters for 2D idealized AMZ model.

Parameter	Symbol	Value	Units
**Phytoplankton growth:**			
Maximum growth rate, P_S	μ_{maxP_S}	0.5	d^{-1}
NO_x^- half-saturation, P_S	$K_{NO_xP_S}$	3.6	nM
NH_4^+ half-saturation, P_S	$K_{NH_4P_S}$	1.8	nM
Chl *a*-specific light absorption, P_S	$a_{P_S}^{chl}$	0.01	m^2 $(mgChl)^{-1}$
Maximum growth rate, P_L	μ_{maxP_L}	3	d^{-1}
NO_x^- half-saturation, P_L	$K_{NO_xP_L}$	327	nM
NH_4^+ half-saturation, P_L	$K_{NH_4P_L}$	164	nM
Chl *a*-specific light absorption, P_L	$a_{P_L}^{chl}$	0.04	m^2 $(mgChl)^{-1}$
Maximum quantum yield	ϕ	0.04	mol C mol^{-1} photons
Chl:C maximum	θ_{max}	0.2	g Chl g^{-1} C
Phytoplankton O_2 production	R_{O_2P}	7.3	mol O_2:mol biomass N
**Grazing and mortality:**			
Grazing coefficient	g	$2\gamma_T$	µM N^{-1} d^{-1}
Grazing efficiency	ζ	0.2	unitless
Grazer O_2 consumption	R_{O_2Z}	7.3	mol O_2:mol biomass N
Oxygen-limiting half-saturation conc. for Z_{P_i}	K_{O_2}	1	µM O_2
Linear mortality rate (B and P)	m_{lin}	$0.01\gamma_T$	d^{-1}
Quadratic mortality rate (B and P)	m_q	$0.1\gamma_T$	µM N^{-1} d^{-1}
Quadratic mortality rate (Z)	m_Z	$0.5\gamma_T$	µM N^{-1} d^{-1}
Fraction of mortality to POM vs. DOM	f_{mort}	0.5	unitless
**Temperature dependence:**			
Reference temperature	T_0	293.15	K
Temperature regulation	A_E	-4000	K
Temperature normalization	τ	0.8	unitless
**Physical parameters:**			
Saturated dissolved oxygen concentration	O_{2sat}	212	µM
Air-sea O_2 transfer coefficient	κ_g	$3 \cdot 10^{-5}$	m s^{-1}
Air-sea equilibration depth	h_g	100	m
Maximum incoming PAR flux	I_{max}	1000	W m^{-2}
PAR attenuation in water	k_w	0.04	m^{-1}
Mixed-layer attenuation depth	z_{ML}	20	m
Horizontal mixing coefficient	κ_X	10^3	m^2 s^{-1}
Minimum vertical mixing coefficient	κ_{Zmin}	10^{-5}	m^2 s^{-1}
Maximum vertical mixing coefficient	κ_{Zmax}	10^{-2}	m^2 s^{-1}
POM sinking rate	w_s	10	m d^{-1}

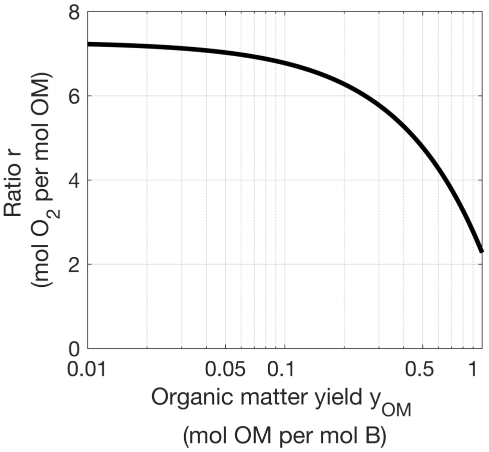

Figure A1: Ratio r (mol O_2 consumed per mol OM consumed) as a function of the average aerobic heterotrophic organic matter yield $y_{OM_{BhetO}}$ mol B synthesized per mol OM consumed) , for $d_{OM} = 29.1$, $d_B = 20$, and with biomass B and organic matter OM accounted for in moles of N. Here, $r \approx 6.6$ mol O_2 per mol organic N, or $r \approx 1$ mol O_2 per mol organic C, reflecting our assumed organic matter stoichiometry. This value is close to the “respiratory quotient” for algal material of 0.9 mol CO_2 produced per mol O_2 (Robinson 2008).

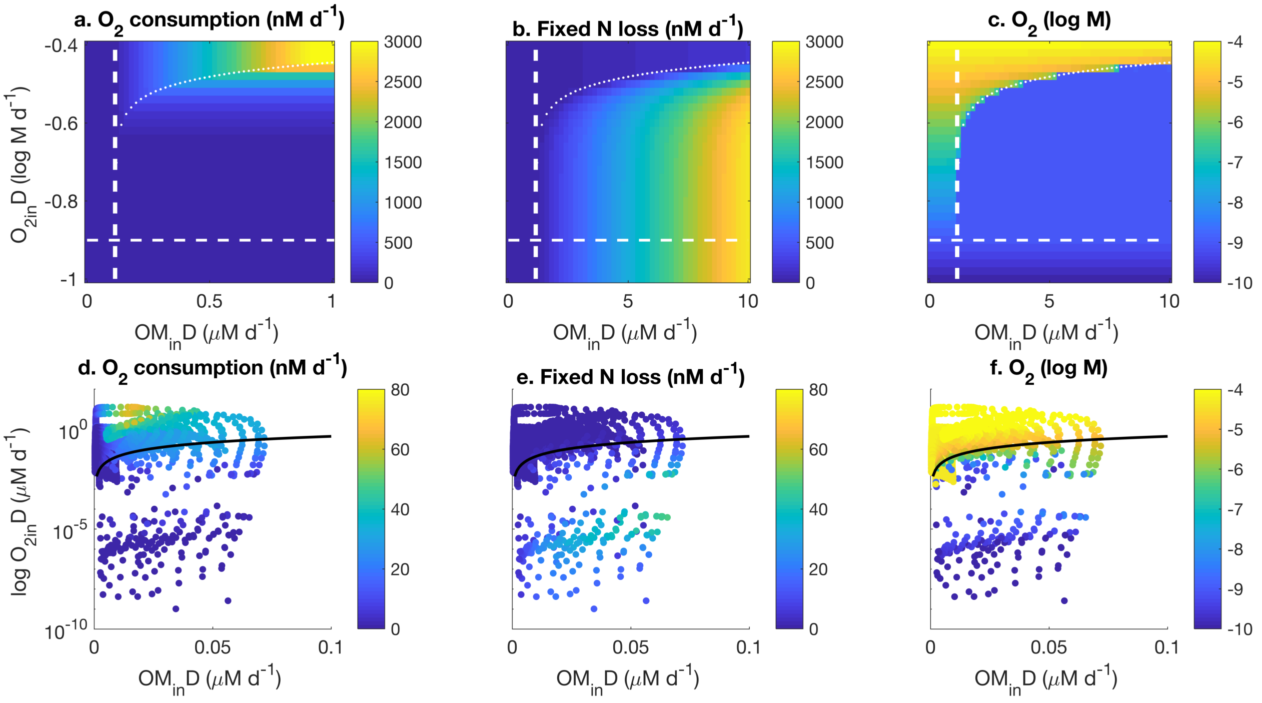

Figure A2: Top: Steady state solutions for varying ratios of oxygen and organic matter supply (incoming concentrations O_{2in} and OM_{in} and dilution rate D) in the virtual chemostat with two metabolisms (a-c), and in the 2D ecosystem model with multiple N-cycling metabolisms (d-f). The curved lines $\phi = 1$ (white lines in a-c, black lines in d-f) indicates the theoretically predicted onset of sustainable coexistence. The dashed, straight white lines in a-c correspond to the subsistence concentrations of oxygen (for the aerobic heterotrophic metabolism) and organic matter (for both aerobic and anaerobic metabolisms; the aerobic population has a slightly lower but visually indistinguishable subsistence concentration). For the 2D model solutions, the incoming oxygen and organic matter supply rates were calculated at each of the 20,000 grid points, and ϕ is calculated accounting for the divergence of the organic matter flux with ϕ_{ocean}.

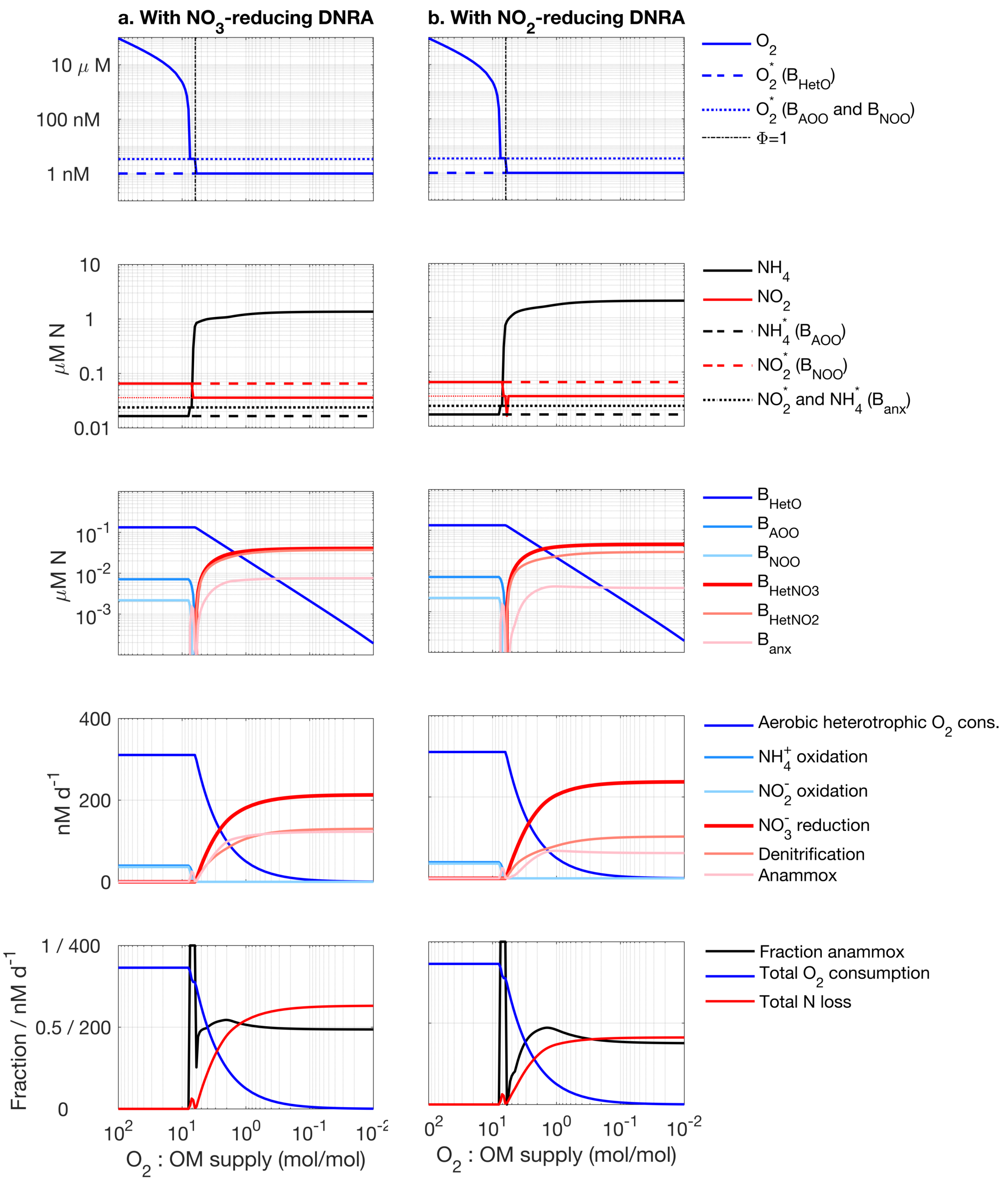

Figure A3: Chemostat model solutions when DNRA (dissimilatory nitrate or nitrite reduction to ammonium) is included as a metabolic functional type. We assumed that the organic matter yield for DNRA was equal to the other anaerobic heterotrophic functional types, allowing for its coexistence in the model with other anaerobic heterotrophs competing for organic substrate in the anoxic state. The type using NO_3^- as an electron acceptor (column a) competed against the NO_3^--reducing heterotroph B_{HetNO_3}, and the type using NO_2^- as an electron acceptor (column b) competed against the denitrifying heterotroph B_{HetNO_2}.

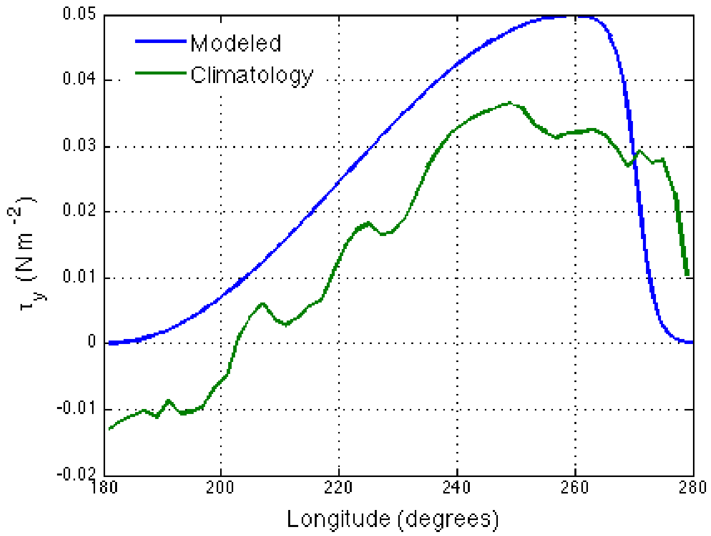

Figure A4: The modeled wind stress used as the forcing for the 2D circulation field against climatological mean. The y component of the annual mean wind stress was averaged meridionally, from the Hellerman and Rosenstein Global Wind Stress Climatology from 180°E to 80°W and 0°S to 10°S (Hellerman and Rosenstein 1983).

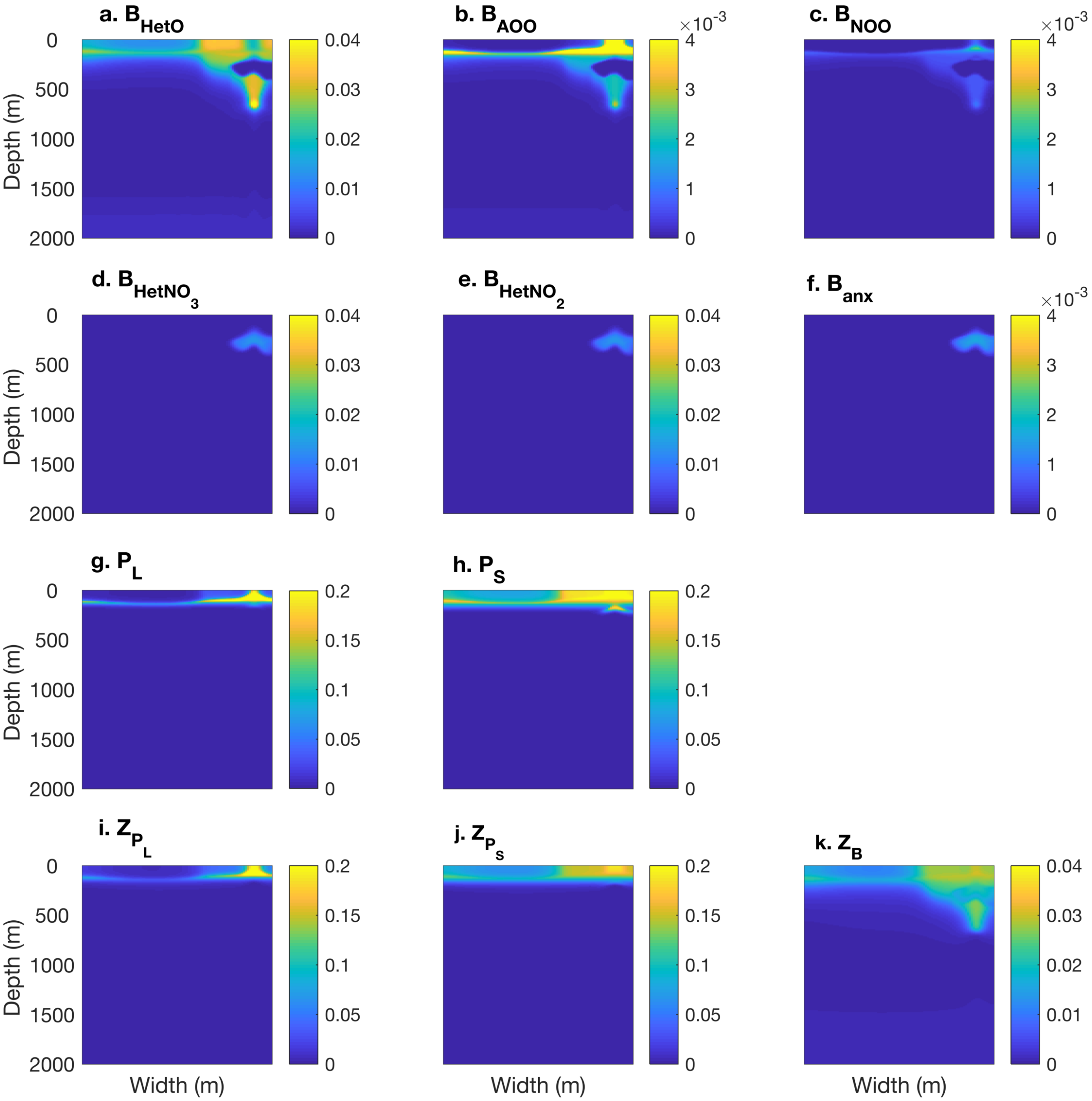

Figure A5: Steady state biomasses of the 11 functional type populations in the 2D model. Units for all: µM N.

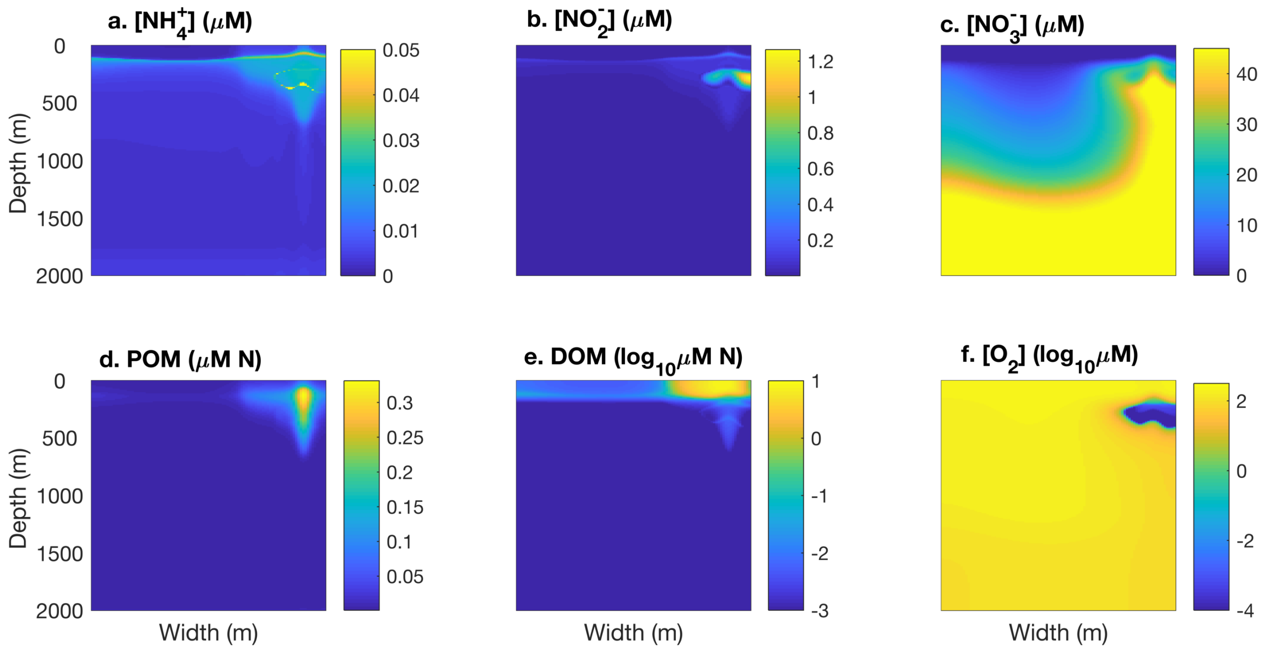

Figure A6: Steady state nutrient and oxygen concentrations in the 2D model.

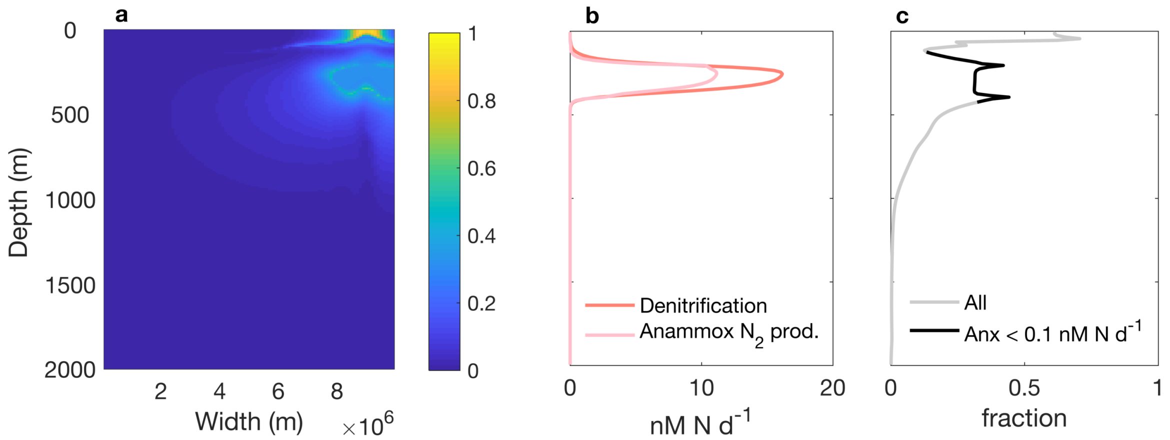

Figure A7: The fraction of anammox contribution to total fixed N loss in the 2D model. The fraction over the whole solution (a) decreases to zero (or increases in a few places) where anaerobic activity is insignificant in the aerobic domain, but this reflects the difference of small numbers. Vertical profiles of the rates of fixed nitrogen loss (b) and the fraction of anammox (c) are illustrated at the same location as in Fig. 5.

References

Anderson, L. A.. 1995. On the hydrogen and oxygen content of marine phytoplankton. *Deep Sea Res. Part I Oceanogr. Res. Pap.*, **42 (9)**, 1675–1680, doi:10.1016/0967-0637(95)00072-E.

Armstrong, R. A.. 1994. Grazing limitation and nutrient limitation in marine ecosystems: Steady state solutions of an ecosystem model with multiple food chains. *Limnol. Oceanogr.*, **39 (3)**, 597–608, doi:10.4319/lo.1994.39.3.0597.

Babbin, A. R., B. D. Peters, C. W. Mordy, B. Widner, K. L. Casciotti, and B. B. Ward. 2017. Novel metabolisms support the anaerobic nitrite budget in the Eastern Tropical South Pacific. *Global Biogeochem. Cycles*, **31**, 1–14, doi:10.1002/2016GB005407.

Bianchi, D., A. R. Babbin, and E. D. Galbraith. 2014. Enhancement of anammox by the excretion of diel vertical migrators. *Proc. Natl. Acad. Sci.*, **111**, 15 653–15 658, doi:10.1073/pnas.1410790111.

Bratbak, G., and I. Dundas. 1984. Bacterial dry matter content and biomass estimations. *Appl. Environ. Microbiol.*, **48 (4)**, 755–7.

Dutkiewicz, S., A. E. Hickman, O. Jahn, W. W. Gregg, C. B. Mouw, and M. J. Follows. 2015. Capturing optically important constituents and properties in a marine biogeochemical and ecosystem model. *Biogeosciences*, **12 (14)**, 4447–4481, doi:10.5194/bg-12-4447-2015.

Escribano, R., P. Hidalgo, and C. Krautz. 2009. Zooplankton associated with the oxygen minimum zone system in the northern upwelling region of Chile during March 2000. *Deep Sea Res. Part II*, **56**, 1049–1060, doi:10.1016/j.dsr2.2008.09.009.

Füssel, J., P. Lam, G. Lavik, M. M. Jensen, M. Holtappels, M. Günter, and M. M. M. Kuypers. 2012. Nitrite oxidation in the Namibian oxygen minimum zone. *ISME J.*, **6**, 1200–9, doi:10.1038/ismej.2011.178.

Geider, R. J., H. L. MacIntyre, and T. M. Kana. 1997. Dynamic model of phytoplankton growth and acclimation: Responses of the balanced growth rate and the chlorophyll a:carbon ratio to light, nutrient-limitation and temperature. *Mar. Ecol. Prog. Ser.*, **148 (1-3)**, 187–200, doi:10.3354/meps148187.

Hellerman, S., and M. Rosenstein. 1983. Normal monthly wind stress over the world ocean with error estimates. *J. Phys. Oceanogr.*, **13**, 1093–1104, doi:10.1175/1520-0485(1983)013⟨1093:NMWSOT⟩2.0.CO;2.

Hickman, A. E., S. Dutkiewicz, R. G. Williams, and M. J. Follows. 2010. Modelling the effects of chromatic adaptation on phytoplankton community structure in the oligotrophic ocean. *Mar. Ecol. Prog. Ser.*, **406**, 1–17, doi:10.3354/meps08588.

Jensen, M. M., P. Lam, N. P. Revsbech, B. Nagel, B. Gaye, M. S. Jetten, and M. M. Kuypers. 2011. Intensive nitrogen loss over the Omani Shelf due to anammox coupled with dissimilatory nitrite reduction to ammonium. *ISME J.*, **5**, 1660–1670, doi:10.1038/ismej.2011.44.

Kalvelage, T., and Coauthors. 2013. Nitrogen cycling driven by organic matter export in the South Pacific oxygen minimum zone. *Nat. Geosci.*, **6**, 228–234, doi:10.1038/ngeo1739.

Lam, P., and Coauthors. 2009. Revising the nitrogen cycle in the Peruvian oxygen minimum zone. *Proc. Natl. Acad. Sci. U. S. A.*, **106 (12)**, 4752–7, doi:10.1073/pnas.0812444106.

LaRowe, D. E., and P. Van Cappellen. 2011. Degradation of natural organic matter: A thermodynamic analysis. *Geochim. Cosmochim. Acta*, **75 (8)**, 2030–2042, doi:10.1016/j.gca.2011.01.020.

Litchman, E., C. A. Klausmeier, O. M. Schofield, and P. G. Falkowski. 2007. The role of functional traits and trade-offs in structuring phytoplankton communities: scaling from cellular to ecosystem level. *Ecol. Lett.*, **10**, 1170–81, doi:10.1111/j.1461-0248.2007.01117.x.

Martens-Habbena, W., P. M. Berube, H. Urakawa, J. R. de la Torre, and D. A. Stahl. 2009. Ammonia oxidation kinetics determine niche separation of nitrifying Archaea and Bacteria. *Nature*, **461 (7266)**, 976–9, doi:10.1038/nature08465.

Penn, J., T. Weber, and C. Deutsch. 2016. Microbial functional diversity alters the structure and sensitivity of oxygen deficient zones. *Geophys. Res. Lett.*, **43**, 9773–9780, doi:10.1002/2016GL070438.

Rittman, B. E., and P. L. McCarty. 2001. *Environmental Biotechnology: Principles and Applications*. McGraw-Hill.

Robinson, C.. 2008. Heterotrophic bacterial respiration. *Microb. Ecol. Ocean.*, D. L. Kirchman, Ed., 2nd ed., Wiley-Blackwell, 299–334.

Strous, M., J. J. Heijnen, J. G. Kuenen, and M. S. M. Jetten. 1998. The sequencing batch reactor as a powerful tool for the study of slowly growing anaerobic ammonium-oxidizing microorganisms. *Appl. Microbiol. Biotechnol.*, **50**, 589–596.

Verdy, A., M. Follows, and G. Flierl. 2009. Optimal phytoplankton cell size in an allometric model. *Mar. Ecol. Prog. Ser.*, **379**, 1–12, doi:10.3354/meps07909.

Ward, B. a., S. Dutkiewicz, O. Jahn, and M. J. Follows. 2012. A size-structured food-web model for the global ocean. *Limnol. Oceanogr.*, **57 (6)**, 1877–1891, doi:10.4319/lo.2012.57.6.1877.

Wishner, K. F., D. M. Outram, B. A. Seibel, K. L. Daly, and R. L. Williams. 2013. Zooplankton in the eastern tropical north Pacific: Boundary effects of oxygen minimum zone expansion. *Deep Sea Res. Part I Oceanogr. Res. Pap.*, **79**, 122–140, doi:10.1016/j.dsr.2013.05.012.

Zakem, E. J., and Coauthors. 2018. Ecological control of nitrite in the upper ocean. *Nat. Commun.*, **9 (1)**, 1206, doi:10.1038/s41467-018-03553-w.

Zimmerman, A. E., S. D. Allison, and A. C. Martiny. 2014. Phylogenetic constraints on elemental stoichiometry and resource allocation in heterotrophic marine bacteria. *Environ. Microbiol.*, **16 (5)**, 1398–1410, doi:10.1111/1462-2920.12329.

Zumft, W. G.. 1997. Cell biology and molecular basis of denitrification. *Microbiol. Mol. Biol. Rev.*, **61 (4)**, 533–616.
